# Hippocampal P2X7 and A2A purinoceptors mediate cognitive impairment caused by long-lasting epileptic seizures

**DOI:** 10.7150/thno.100365

**Published:** 2025-02-10

**Authors:** Meng-Juan Sun, Wen-Jing Ren, Ya-Fei Zhao, Xuan Li, Muhammad Tahir Khan, Xin-Yi Cheng, Hai-Yan Yin, Alexei Verkhratsky, Tobias Engel, Patrizia Rubini, Yong Tang, Peter Illes

**Affiliations:** 1 International Joint Research Centre on Purinergic Signalling, School of Acupuncture and Tuina, Chengdu University of Traditional Chinese Medicine, Chengdu, China.; 2 Faculty of Biology, Medicine and Health, The University of Manchester, Manchester, UK.; 3 Department of Physiology and Medical Physics, Royal College of Surgeons in Ireland, University of Medicine and Health Sciences, Dublin, Ireland.; 4 FutureNeuro, Science Foundation Ireland Research Centre for Chronic and Rare Neurological Diseases, Royal College of Surgeons in Ireland, University of Medicine and Health Sciences, Dublin, Ireland.; 5 Acupuncture and Chronobiology Key Laboratory of Sichuan Province, Chengdu, China.; 6 School of Health and Rehabilitation, Chengdu University of Traditional Chinese Medicine, Chengdu, China.; 7 Rudolf Boehm Institute for Pharmacology and Toxicology, University of Leipzig, Leipzig, Germany.

**Keywords:** hippocampus, status epilepticus, ATP, adenosine, P2X7 receptors, A2A receptors, pharmacological antagonists

## Abstract

**Rationale:** Cognitive impairment and depression are salient comorbidities of mesial temporal lobe epilepsy; it is still unclear whether this frequently drug resistant disease is a cause or consequence of hippocampal damage and its interplay with long-lasting seizure activity (*status epilepticus*; SE). Thus, a major therapeutic advance in this field is badly needed.

**Methods:** We modeled enduring behavioral and electroencephalographic (EEG) seizures in mice by the intraperitoneal injection of kainic acid (KA), and measured the dynamics of the intracellular Ca^2+^ signals in the hippocampal CA1 area by fiber photometry. Learning and memory were controlled by the Morris Water-Maze and Novel Object Recognition tests on whole animals and by the induction of long-term potentiation in CA1 pyramidal neurons in brain slices. Depressive-like reactions were evaluated by the Tail Suspension, Forced Swim, and Sucrose Preference tests.

**Results:** The intraperitoneal injection of the blood-brain permeable, highly selective, P2X7 and A2A receptor (R) antagonists, JNJ-47965567, and KW6002/SCH58261, respectively, counteracted the effects of KA-induced SE both on seizure activity and the increase of Ca^2+^ signals (as a measure of changes in the intracellular Ca^2+^ concentration) in neurons and astrocytes of the hippocampal CA1 area. In addition, these drugs also prevented the impairment of the hippocampus-dependent spatial and non-spatial learning abilities by KA-SE. The knockdown of P2X7Rs in CA1 astrocytes, but not neurons prevented the cognitive deterioration, suggesting that the release of astrocytic signaling molecules onto neighboring neurons might be the cause of this effect. In accordance with our observations, in hippocampal slices prepared from mice which underwent KA-SE, a selective sensitivity increases to the prototypic P2X7R agonist dibenzoyl-ATP (Bz-ATP) manifested in CA1 neurons. This sensitivity increase appeared to be due to a postsynaptic interference between P2X7Rs and the release of excitatory neurotransmitters during SE. In spite of a P2X7 and A2AR-mediated increase of Ca^2+^ signaling in the medial prefrontal cortex, no similar change was noted after KA-SE in depressive-like reactions or the open-field behavior.

**Conclusions:** SE induced the release of ATP and adenosine from the hippocampus and in consequence decreased the cognitive abilities of mice. The pharmacological blockade of P2X7 and A2ARs prevented the SE-induced seizure activity and cognitive deterioration, but not depressive-like behavior.

## Introduction

Epilepsy is one of the most common neurological diseases affecting the CNS with an incidence of 1-2% within the general population, occurring especially in the young and elderly 1,2. It is characterized by spontaneous seizures caused by hyperexcitability of neurons in the cerebral cortex due to imbalance of synaptic excitation and inhibition; epilepsy can result from genetic abnormalities or can be acquired following a precipitating injury 3,4. *Status epilepticus* (SE), associated with significant morbidity and mortality, is defined as a continuous seizure lasting more than 5 min (previously by definition 30 min) or when seizures occur closely together without allowing the patients to return to a normal level of consciousness between episodes; it reflects the failure of mechanisms normally terminating seizures 5.

In the CNS, purines, such as ATP and its enzymatic degradation products ADP and adenosine, or UTP and its enzymatic breakdown product UDP, are released into the extracellular space from neuronal and non-neuronal cells by a variety of mechanisms and function as signaling molecules subserving cell-to-cell communication 6,7. The term 'purinome' describes the entity of the nucleotides, nucleosides, the membrane receptors on which they act, and the degrading/transforming enzymes, and uptake mechanisms (only for adenosine) as a functional and mutually interacting unit 8.

Increased quantities of ATP have been shown to be released from human hippocampal slices of drug-resistant epilepsy patients on stimulation by high K^+^ medium, causing ictal discharges in tissue from the epileptic focus 9,10. In addition, kainic acid (KA)-induced convulsions triggered an increased ATP release from synaptic preparations of the mouse hippocampus, when compared with the corresponding preparations of vehicle treated mice 11. In the context of epilepsy, two purinoceptors, the ionotropic ATP receptor P2X7 and the metabotropic adenosine receptors A1 and A2A are of special importance.

P2X7Rs are expressed at the highest density at microglial cells, the resident macrophages of the brain and spinal cord, and their stimulation by large concentrations of ATP leads to the production and subsequent secretion of inflammatory cytokines/chemokines and favors the production of reactive oxygen/nitrogen radicals as well as of proteolytic enzymes 12,13. All these effects contribute to the necrotic/apoptotic properties of P2X7Rs 14. In consequence, selective P2X7R antagonists have beneficial effects in a range of neurodegenerative illnesses, such as neuropathic pain, Alzheimer's disease, Parkinson's disease, and multiple sclerosis, but also in neurodegeneration following SE 15,16.

Adenosine receptors have two divergent and opposite effects on epilepsy. Inhibitory A1Rs located presynaptically at excitatory nerve terminals decrease transmitter release, whereas at postsynaptic sites at neuronal cell bodies they cause hyperpolarization, and by both modes of action downregulate neuronal excitability 17,18. By contrast, selective A2AR agonists acting on receptors situated at glutamatergic nerve terminals increase the release of the excitatory/excytotoxic glutamate thereby promoting neurodegeneration 17. The blockade of A2ARs attenuates the severity of epileptic seizures, and prevents neuronal damage following convulsions 19,20.

Epilepsy associates with several well-known co-morbidities, including anxiety-depression and cognitive deterioration, which exert a substantial limitation to the quality of life of the afflicted patients 21. It is no wonder that these co-morbidities prevail after long-lasting epilepsy because they share the etiological factors including neuroinflammation and/or accompanying neurodegeneration with SE.

Our aim was to investigate the chain of events in the hippocampal CA1 region initiating these pathological events in a model of KA-induced SE in the mouse 22. We found that although indirect evidence is compatible with the participation of P2X7 and especially A2ARs in these events, the general picture is still quite blurred and needs a consequent clarification. In conclusion, we report the novel findings, that KA-induced SE causes the increase of Ca^2+^ signals (as a measure of changes in the intracellular Ca^2+^ concentration; [Ca^2+^]_i_) in hippocampal CA1 neurons and astrocytes of mice. This leads to a consequent ATP and adenosine release, which activates local P2X7 and A2ARs, respectively, resulting in cognitive deterioration, but no depressive-like behaviors.

## Methods

### Animals

Juvenile (3-4 weeks old; patch clamp recordings) and adult mice (6-8 weeks old; all other experiments) were housed under standard laboratory conditions (24 ± 2°C room temperature and 65 ± 5% humidity on 12/12 h light-dark cycles) with drinking water and food available *ad libitum*. C57BL/6J male mice (from Chengdu Dossy Experimental Animal Co., Chengdu, China) were used for all experiments and their controls reported in this paper, except when 8-10 weeks old P2X7R knock out (KO) (P2rx7^-/-^) mice (6NTac;B6N-P2rx7tm1d [EUCOMM]Wtsi/Ieg; 23, and their wild-type (WT) controls of the same age (C57/Bl6 OlaHsd) were utilized for the adult neural progenitor cell (NPC) measurements. The animal study was reviewed and approved by the Institutional Review Board of the Chengdu University of Traditional Medicine, Chengdu, China (protocol code, DC1647, 12 February, 2020).

### Epilepsy models and EEG monitoring

KA (30 mg/kg) was injected intraperitoneally (i.p.). The dose of KA and its route of application were the same in all experiments included in this paper. The mice in the saline group were injected with normal saline instead of KA. A modified Racine scale for the measurement of the intensity of seizures was as follows 24: 0, complete absence of motor convulsions; 1, stupor, closed eyes, seizures of ears and whiskers, sniffing, facial clonus; 2, nodding because of a stronger facial clonus; 3, clonus of bilateral forelimbs without rearing; 4, bilateral forelimbs clonus with rearing; 5, falling on the side (without rearing), loss of setup reflex, paralleled by generalized clonic convulsions. Seizures at stages 4-5 that last for ≥30 min were defined as SE.

The mortality of mice was 10-15% in our experiments with 30 mg/kg, i.p. KA, and thereby comparable to that reported by Augusto 11 (35 mg/kg KA, subcutaneously; s.c.). The mortality could have been reduced by the i.p. injection of diazepam, 1 h after injection of KA, to terminate seizures (e.g. 25), although of course death during the 1 h period is even in this case unavoidable. Moreover, treatment with the sedative/antiepileptic diazepam and the subsequent measurement of cognitive abilities certainly affected by sedation, is not compatible with the present experimental aims. Hence, we relinquished from the application of diazepam, because the mortality was relatively low and death within the 1-h period before diazepam injection could not have been avoided either. The onset to the first seizure of stages 4 or 5, and the duration of these seizures were calculated.

The mice were anesthetized with isoflurane (5% induction; 2% maintenance; RWD Life Science, San Diego, CA, USA) and fixed on a stereotaxic platform (RWD Life Science). Ear bars and a mouth holder were used to keep the mouse head in place while the skin was shaved and disinfected with a povidone/iodine solution. The skull was exposed and 1-mm-diameter craniotomy was made with a microdrill mounted to the stereotaxic manipulator. Three screws with EEG recording wires were implanted into the right frontal bone and bilateral suboccipital bone and fixed with dental cement. At the end of the surgery, 5 mg/kg enrofloxacin (RWD Life Science) was administered subcutaneously (s.c.) to the animals to prevent postoperative infection, and all of the mice were placed on heating pads (37 °C) during surgery to keep their body temperature stable.

At least 2 weeks were allowed to pass before starting an experimental measurement. EEG signals were recorded by a telemetric apparatus (PowerLab; AD Instruments, Bella Vista, Australia) and evaluated by the software LabChart 8 (AD Instruments, Colorado Springs, CO, USA), during KA-induced status epilepticus and time matching saline-injected controls. The EEG signals were recorded for the duration of 1 h, starting 1 h after the injection of KA. In order to quantify the EEG activity, the n-fold increase of the total power was presented in each case. EEG total power (µV^2^) is a function of EEG amplitude over time and was analyzed by integrating frequency bands from 0 - 100 Hz. Power spectral density bands were generated within the LabChart 8 (spectral view), with the frequency domain filtered from 0 - 40 Hz and the amplitude domain filtered from 0 - 50 mV. For calculation of the n-fold total power also the LabChart 8 was used and for greater uniformity the period between 32-36 min was selected.

### Stereotaxic surgery and viruses

Injections of viruses were performed after EEG electrode implantation under the same anesthesia as described previously. For virus injection a glass pipette was mounted in a Nanoject 3 infusion system (Drummond Scientific, Broomail, PA, USA). Then, 300 nl of each virus was infused over 10 min into the left hippocampal CA1 area, and the left medial prefrontal cortex (mPFC) with the stereotactic co-ordinates, AP = -1.85 mm, ML = -2.15 mm, DV = -1.6 mm, and AP = 1.78 mm, ML = -0.35 mm, DV = -2.25 mm, respectively. The glass pipettes used for infusion were left in place for at least 5 min.

The adeno-associated viruses (AAVs) used were rAAV-hSyn-GCaMP6f-EGFP (generates in neurons the genetic Ca^2+^ indicator), and rAAV-GfaABC1D-GCaMP6f-EGFP (generates in astrocytes the Ca^2+^ indicator; 26; Brain Case; Wuhan, China). In separate experiments, the synthesis of a GRAB sensor detecting ATP was induced by the injection of 300 nl of rAAV-hSyn-ATP1.0 or its control, rAAV-hSyn-ATP1.0mut 27 into the left hippocampal CA1 area at the above indicated stereotactic coordinates, as well as the synthesis of another GRAB sensor detecting adenosine was induced by the injection at the same site of rAAV-hSyn-Ado1.0med, or its control, rAAV-hSyn-Ado1.0mut 28 (Brain Case).

To knock down murine P2X7R (mP2X7R) expression in neurons and astrocytes, AAVs expressing shRNA directed against mP2RX7 (5´-GCGGAAAGAGCCTGTTATCAG-3´) or control shRNA which are driven by cell type-specific promoters were injected. The following viruses were generated by Guangzhou PackGene Biotechnology Co., Ltd. (Guangzou, China): Neuron-specific (driven by human synapsin promoter): AAV9_hSyn-EGFP-miR30shRNA(mP2X7)-WPRE-SV40pA and AAV9_hSyn-EGFP-miR30shRNA(NC)-WPRE-SV40pA as well as astrocyte-specific (driven by 681 bp GFAP promoter): AAV8_GFAP(681bp)-EGFP-miR30shRNA(mP2X7)-WPRE-SV40pA and AAV8_GFAP(681bp)-EGFP-miR30shRNA(NC)-WPRE-SV40pA. Cell-specific shRNAs were injected in a volume of 3 µl by a 5 µl microsyringe, inserted into the right lateral ventricle at the following stereotactic co-ordinates (AP = -0.4 mm, ML = 1.0 mm, DV = 3.0 mm; 29,30. At the end of surgery, 5 mg/kg enrofloxacin (RWD Life Science) was administered s.c. to the animals to prevent postoperative infection. All mice were placed on heating pads (37°C) during surgery to keep their body temperature stable. Experiments were made 2 weeks after micro-injecting the viruses.

### *In vivo* fiber photometry recording

**Figure 1:**
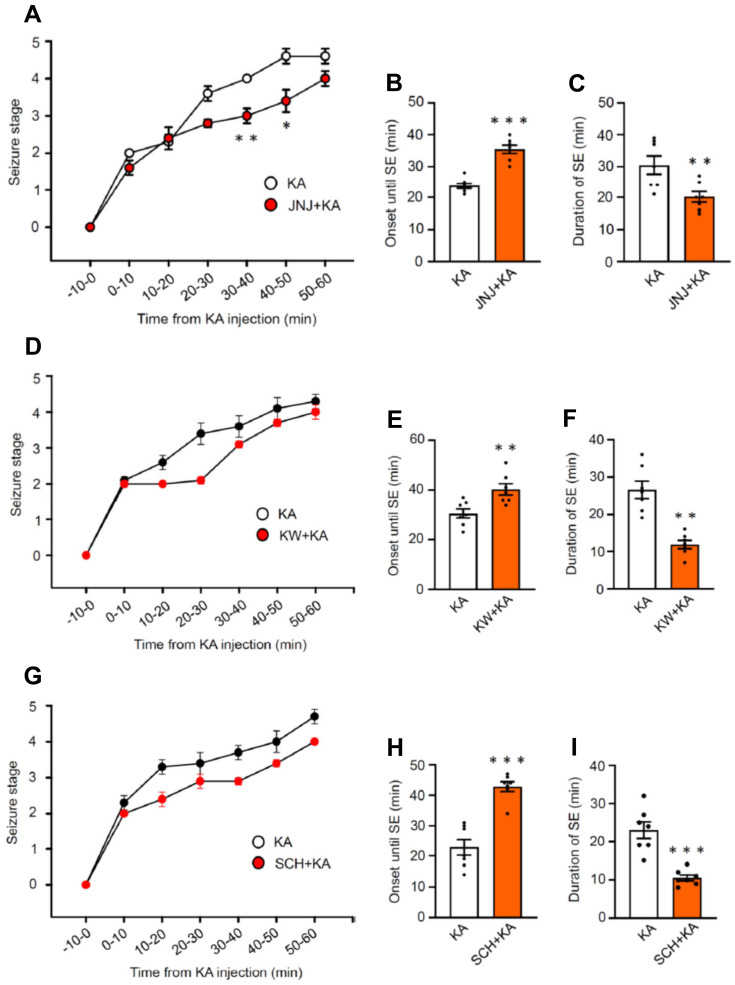
Seizure stages on the Racine scale in mice during the first 1 h after the injection of kainic acid (30 mg/kg) or its application together with a preceding injection of JNJ-47965567 (30 mg/kg), KW6002, or SCH58261 (3 mg/kg each), all via the i.p. route. While the curve of the seizure stage was displaced to the right by JNJ-47965567, it was unchanged by KW6002 and SCH58261.The Mann-Whitney test was used for statistical evaluation; the levels of significance reached were *P < 0.05, **P < 0.01 (**A,** 30-40 min, U = 3.500, P = 0.0202; **B,** 40-50 min, U = 6, P = 0.0204; **D,** 30-40 min, U = 15, P = 0.2692; **G,** 40-50 min, U = 17, P = 04371). In addition, we also calculated the onset until SE (stages 4-5) and the duration of SE, which were in all cases modified by JNJ-47965567, KW6002, and SCH58261. The statistical evaluation with the unpaired t-test yielded the following results: *P < 0.05, **P < 0.01, *P < 0.001 (**B,** t = 7.475; **C,** t = 3.093; **E,** t = 6.483; **F,** t = 5.409; **H,** t = 3.277; **I,** t = 5.734 (n = 7 each).

**Figure 2:**
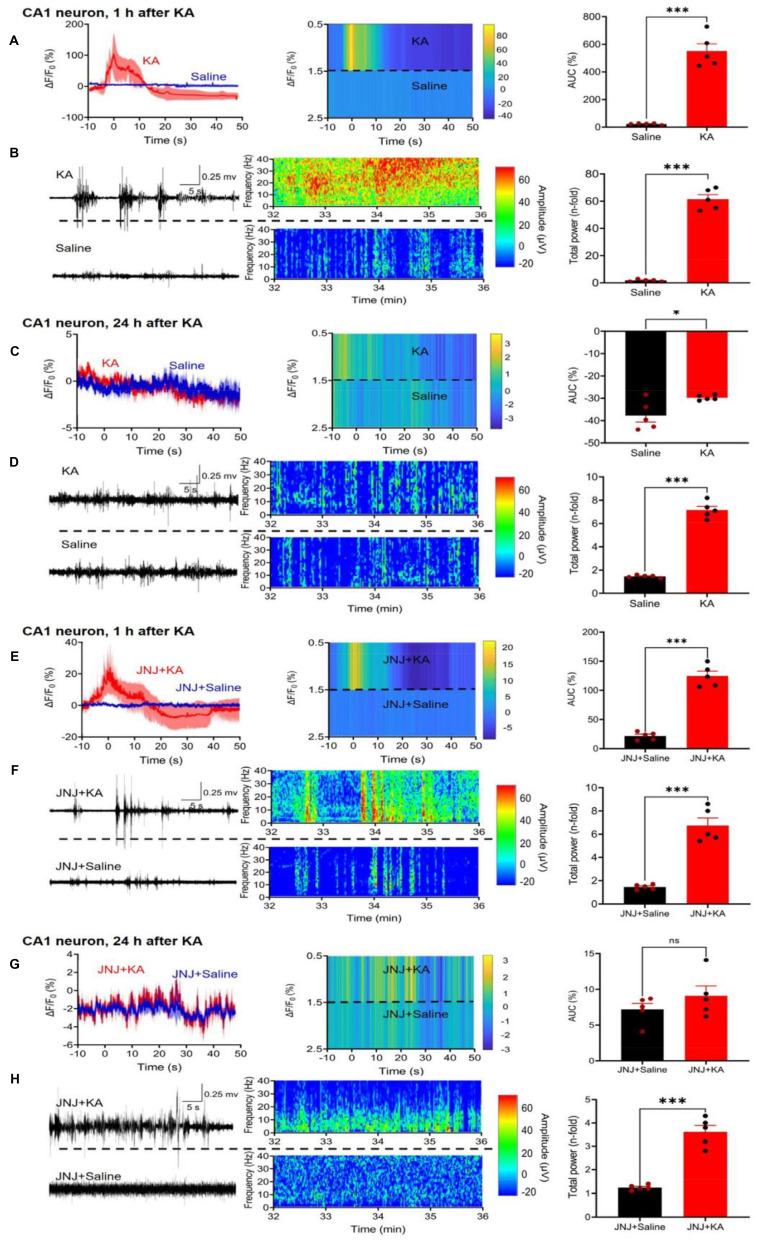
Effect of kainic acid (KA) injection alone or in combination with the P2X7R antagonist JNJ-47965567 on the intracellular concentration of Ca^2+^ in CA1 neurons of the mouse hippocampus. Two weeks before KA application (30 mg/kg, i.p.), the virus complex rAAV-hSyn-GCaMP6f-EGFP generating specifically in neurons a genetic Ca^2+^ indicator was microinjected into the hippocampus. JNJ-47965567 (30 mg/kg, i.p.) was injected 1 h before KA. The Ca^2+^ changes were measured with fiber photometry and expressed in ΔF/F_0_; gross electrical activity of the brain was measured with EEG telemetry and presented in µV amplitude signals (left panels). The ΔF/F_0_ fluorescence curves are shown as mean ± S.E.M. of the recordings occurring during the seizure stages 4 and 5. KA was dissolved in saline, while JNJ-47965567 was dissolved in 30% SBE-β-CD + 70% saline (the solvent of JNJ-4438079 is indicated in the Figure panels as saline for the sake of simplicity). All details of the procedures used are described in the Methods Section. The heatmaps of the Ca^2+^ and EEG recordings are shown in the middle panels, while the right panels show the area under the curve (AUC) of the ΔF/F_0_ changes and the total power of the EEG as n-time changes from the baseline. The levels of statistical significance reached are marked with *P < 0.05, **P < 0.01, and ***P < 0.001. The unpaired t-test was used for evaluation in the case of normal distribution of data (**A**, t = 10.00; **C**, t = 2,685; **D**, t = 6,517; **E**, t = 11.69; **F**, t = 8.031; **G**, t = 1.189; **H**, t = 8.577). The Mann-Whitney test was used for evaluation in panel **B** because of non-normal distribution of the data (U = 0). The number of experiments was 5 in each panel.

**Figure 3:**
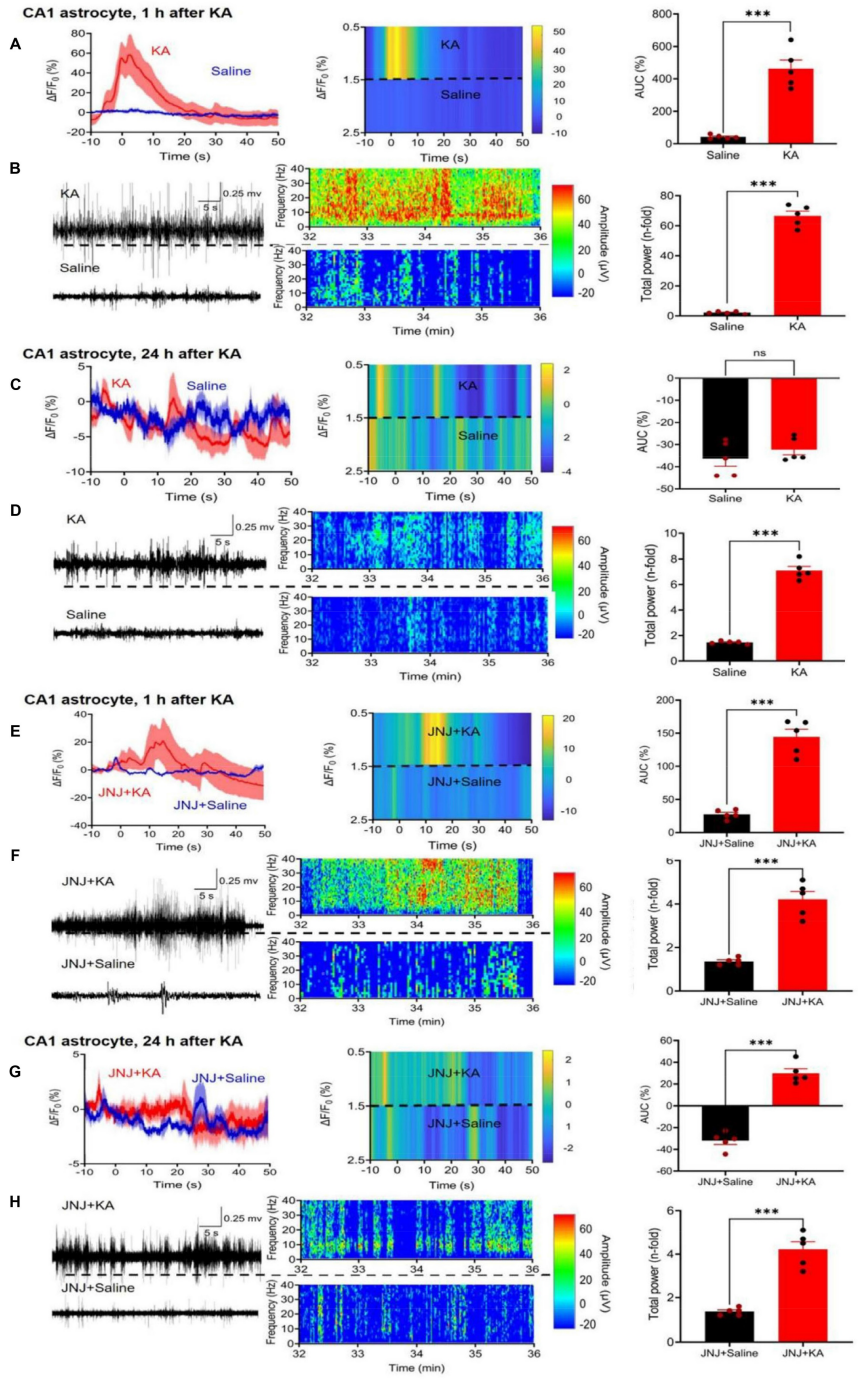
Effect of kainic acid (KA) injection alone or in combination with the P2X7R antagonist JNJ-4438079 on Ca^2+^ signals in CA1 astrocytes of the mouse hippocampus. Two weeks before KA application (30 mg/kg, i.p.), the virus complex rAAV-GfaABC1D-GCaMP6f-EGFP generating specifically in astrocytes a genetic Ca^2+^ indicator was microinjected into the hippocampus. JNJ-47965567 (30 mg/kg, i.p.) was injected 1 h before KA. The changes in Ca^2+^ signaling was measured with fiber photometry and expressed in ΔF/F_0_; gross electrical activity of the brain was measured with EEG telemetry and presented in µV amplitude signals (left panels). The ΔF/F_0_ fluorescence curves are presented as mean ± S.E.M. of the recordings occurring during the seizure stages 4 and 5. KA was dissolved in saline, while JNJ-47965567 was dissolved in 30% SBE-β-CD + 70% saline (the solvent of JNJ-4438079 is indicated in the Figure panels as saline for the sake of simplicity). All details of the procedures used are described in the Methods Section. EEG recordings are shown in the middle panels, while the right panels show the area under the curve (AUC) of the ΔF/F_0_ changes and the total power of the EEG as n-time changes from the baseline. The levels of statistical significance reached are marked with *P < 0.05, **P < 0.01, and ***P < 0.001. The unpaired t-test was used for evaluation throughout (**A**, t = 7.809; **B**, t = 20.26; **C**, t = 0.9751; **D**, t = 17.52; **E**, t = 9.273; **F**, t = 11.20; **G**, t = 11.20; **H**, t = 7.902). The number of experiments was 5 in each panel.

Population calcium signals were collected with a commercialized fiber photometry system (ThinkerTech Inc., Singapore; 31). At 14 days before recording, an optic fiber coated with ceramic ferrule (diameter: 1.25 mm, Thinker Tech Inc.) was implanted into the CA1 region of the hippocampus (stereotactic co-ordinates: AP: -1.85 mm; ML: -2.15 mm; DV: -1.58 mm). The base of the optical fiber was fixed with a screw onto the skull. During recording, a 470 nm LED (40 mW at fiber tip) was used for excitation, while calcium independent signals were obtained using a 410 nm LED (20 mW at fiber tip) to correct for movement artifacts. The fluorescence signals were filtered (35 Hz cut-off) and normalized to calculate the fluorescent change (ΔF/F_0_), where F_0_ was the baseline fluorescent level. Fluorescent signals were recorded for the duration of 1 h, starting 1 h after the injection of KA. For behavioral recording, each event trace was extracted with reference to the tag (from -10 s to +50 s); the mean ± SEM of each ΔF/F_0_ curve is shown in the respective Figures (1 h after KA injection, in the absence of any receptor antagonist, only curves accompanied by Racine 4-5 seizures were included into evaluations). We have chosen for area under the curve (AUC) calculation only the second half of the curve, because the first half started quite variably somewhere between -10 and 0 s (see e.g. Figure 2A and E, or Figure 3A and E). Relative fluorescence was converted to the Z-score and presented as heatmap series. The intensity of the fluorescence signal was calculated between 0 and 20 s, and expressed as AUC by using the MATLAB 2017B program (Mathworks, Natick, MA, USA). This procedure is only an approximation, especially because not the whole AUC is taken into consideration, but has the advantage to introduce some standardization into the calculations.

### Open field test

The apparatus consisted of a rectangular chamber (50 × 50 × 50 cm) made of white, high density, non-porous plastic. The mice were gently placed in the center of the chamber and their motility was recorded for 10 min. The total running distance, and the time spent in the center versus the periphery of the open field chamber were recorded by a camera connected to a computer using an automated video tracking program (EthoVision XT 9.0; Noldus, Wageningen, The Netherlands). The chamber was thoroughly cleaned with 95% ethanol, and dried prior to use before subsequent tests, to remove any scent clues left by the previous subject.

### Tail suspension test (TST)

Mice were suspended on a 55 cm high laboratory rack by adhesive tape positioned about 1 cm from the tail tip. The approximate distance between the animal's nose and the operating floor was 20-25 cm. The animals were separated from each other by baffles to prevent mutual interference during the 6 min suspension time. The cumulative immobility time was recorded in each case. The TST was performed 10 min after injection of KA alone or in combination with P2X7R or A2AR antagonists, and only a single time for each animal. All of the behavioral tests were made at roughly the same time of the day (10:00 a.m. to 12:00 a.m.) and the evaluator was blind to the mice conditions.

### Forced swim test (FST)

The FST was performed in a clear glass cylinder filled with water (temperature, 23-25^o^C). The volume of the cylinder was the following: height, 30 cm; diameter, 20 cm; and water level, 15 cm. The mice were placed gently in the tanks. The duration of immobility within the 6 min of observation was determined. The movement of the animals was video recorded and analyzed later. Following the swimming sessions, the mice were removed from the water by their tails, gently dried with towels and kept warm under a lamp in their home cages. They were considered to be immobile, whenever they stopped swimming and remained passively floating still keeping their heads above the surface of the water. The FST was performed 24 h after injection of KA alone or in combination with P2X7R or A2AR antagonists, and only a single time for each animal.

### Sucrose preference test (SPT)

The mice were singly caged for 3 days and given two 50 ml bottles of water and a 1% sucrose solution (wt/vol), respectively. The bottle positions were switched daily to avoid a side bias. Following a 22-h period of water and food deprivation, the preferences for sucrose or water was determined overnight. Sucrose preference (%) was quantified as (vol sucrose)/(vol sucrose + vol water) x 100%. KA alone or in combination with P2X7R or A2AR antagonists, and only a single time for each animal, was injected 24 h before the 3 training days.

### Morris water maze (MWM) test

The MWM test was conducted in a circular tank (diameter: 90 cm; height: 30 cm) in a dimly lit room. The water temperature was kept at 22-25^o^C somewhat below the temperature pleasant enough to tempt the mice to float. A removable circular platform (diameter, 9.5 cm; height, 28 cm) was positioned 1.5 cm below the opaque water surface (non-fat milk was added for this purpose) in one of the quadrants. The pool area was divided into four equal quadrants, and markers were added in different shapes by affixing color cards. During the 4 days of visible platform training, each mouse received 4 training trials every day. On the 5^th^ day (1 d after the injection of KA), all mice were allowed to make one probe trial for 60 s of searching after removing the platform from the tank. The time spent by the mice in each quadrant was measured; in the meantime, total swimming distance, escape latency, duration in target quadrant and crossing time in each quadrant were automatically recorded by the water maze system (Chengdu Techman Software Co., Inc., Chengdu, China).

### Novel object recognition test (NORT)

Data were collected using an open field test box (40 × 40 × 35 cm). The NORT test was performed during three days. On the first day, each mouse was allowed to explore freely a rectangular arena for 10 min. On the second day, two identical items were placed in the arena. Each mouse was allowed again to explore the arena for 10 min. When the second day's experiment was finished, mice were injected with KA. On the third day (1 d after the injection of KA), one of the objects was replaced with a different one. The explorations were recorded by a night vision camera (ELP1 Megapixel Day Night Vision; Inf iRay, Shenzen, China) and the exploration processes were analyzed with SMART 3.0 video tracking software (Panlab, Barcelona, Spain). The novel object recognition ratio was analyzed as a percentage ratio of the time spent exploring the novel object over the time-period of 5 min.

### Immunofluorescence staining

For visualizing the co-localization of the neuronal marker NeuN or the astrocytic marker GFAP with enhanced green fluorescent protein (EGFP) of the AAVs, the mice were sacrificed 2 weeks after AAV application with the i.p. injection of 1% sodium pentobarbital (0.4 ml; Sigma-Aldrich, St. Louis, MO, USA) and were then transcardially perfused with normal saline followed by pre-cold paraformaldehyde (4% w/v in phosphate buffered saline; PBS). The brains were removed and postfixed in 4% paraformaldehyde overnight. Subsequently, they were dehydrated with gradient (20-30%) sucrose in PBS at 4^o^C. Coronal 40 μm-thick sections were prepared and incubated in a cryostat (Leica CM1860, Leica Biosystem, Muttenz, Switzerland) at -20°C until use. The sections were afterwards incubated in a blocking solution containing 4% bovine serum albumin (Sigma-Aldrich), and 0.5% Triton X-100 (Solarbio, Beijing, China) for 2 h at room temperature. Afterwards, the sections were incubated with mouse anti-GFAP (1:500; Cell Signalling Technology, Dalton, MA, USA) and mouse anti-NeuN (1:1000; Abcam, Cambridge, MA, USA) overnight at 4°C in the blocking solution. After washing with 0.1 M PBS containing 0.1% Triton X-100, the sections were incubated for 2 h with goat anti-mouse AF594 secondary antibodies (1:200, Bioss, Beijing, China) and DAPI (1:1000, Abmole Bioscience, TX, USA) in the blocking solution at room temperature, washed three times with 0.1 M PBS containing 0.1% Triton X-100 and then mounted on slides using coverslips. Image acquisition was performed using a confocal laser scanning microscope (Olympus IXplore SpinSR, Olympus, Tokyo, Japan).

A slightly different procedure was used for hippocampal specimens used to evaluate doublecortin (DCX)-immunoreactive adult neural progenitor cells (NPCs) of the subgranular zone of the dentate gyrus in the hippocampus of wild-type and P2X7R KO mice, 3 weeks after the injection of saline or KA i.p. Following 24 h of post-fixation in 4% PFA at 4°C, brains were transferred to PBS and immersed into 4% agarose. 50 µm sagittal sections were cut using the VT1000S vibratome (Leica Biosystems, Wetzlar, Germany) and sections were stored at -20°C in glycol. Here, brain slices first underwent a membrane permeabilization step, followed by an antigen-retrieval step with hydrochloric acid. Tissue sections were incubated with 0.1% triton/PBS, followed by 1 M glycine and with 1% BSA-PBS. Sections were then incubated with a primary antibody (rabbit anti-doublecortin; 1:800, Cell Signalling Technology) overnight. After that, sections were rinsed and incubated with the secondary Alexa-conjugated antibody (1:1000) for 2h, AlexaFluor-488 goat anti-rabbit (LifeTechnologies, Waltham, MA, USA), followed by a short incubation with DAPI (1:500; Sigma-Aldrich). FluorSave™ (Millipore, Dublin, Ireland) was used to mount tissue slices. Microscope images were taken with a Leica DM400b microscope equipped with two laser lines (405 and 488nm) using a 10x and 40x immersion objective and Image J software.

### Patch clamp recordings

C57BL/6J mice, 3-4 weeks old, and thereby younger than mice used in the rest of the experiments, were utilized. Ample evidence shows that patch-clamp recordings from neurons (but to a minor extent also astrocytes) becomes increasingly difficult with advanced age). The preparation of hippocampal brain slices and patch-clamp procedures were as described previously 32,33. In short, after decapitation, the brain was placed into ice-cold, oxygenated (95% O_2_ + 5% CO_2_) artificial cerebrospinal fluid (aCSF) of the following composition (in mM): NaCl 126, KCl 2.5, CaCl_2_ 2.4, MgCl_2_ 1.3, NaH_2_PO_4_ 1.2, NaHCO_3_ 25, and glucose 11. Then, hippocampal slices were cut at the thickness of 200 μm by using a vibratome (VT1200S; Leica Biosystems, Muttenz, Switzerland) and slices were then transferred to a room temperature holding chamber for at least 1 h.

To create low divalent cationic conditions (low X^2+^) in experiments when NMDA and BzATP were applied to neurons and astrocytes (Figure 9A-F), MgCl_2_ was omitted from the medium and the CaCl_2_ concentration was decreased to 0.5 mM. The hippocampal slices were transferred from the holding chamber to a submerged organ bath kept at room temperature (20-24^o^C) and superfused with 95% O_2_+5% CO_2_-saturated low X^2+^ aCSF. Neurons and astrocytes in the CA1 region were visualized by using a 40x water immersion objective (LUMPlanFLN; Olympus, Tokyo, Japan). Patch pipettes were filled with an intracellular solution of the following composition (in mM): K-gluconic acid 140, NaCl 10, MgCl_2_ 1, HEPES 10, EGTA 11, Mg-ATP 1.5, Li-GTP 0.3; pH 7.3; 290-310 mOsm. The pipette resistance with these solutions and in normal aCSF was 3-5 MΩ. The series resistance was continuously monitored using 100-ms long -5 mV voltage steps and experiments were discarded when the change in series resistance surmounted 10% at the end of recordings.

Whole cell current-clamp and voltage-clamp recordings were made with a patch-clamp amplifier (MultiClamp 700B; Molecular Device, San Jose, CA, USA). CA1 astrocytes were discriminated from neurons by their failure to fire action potentials in response to supra-threshold depolarizing current injection in the current-clamp mode of the amplifier. Almost all non-spiking cells belonged to the passive class (typical astrocytes) and only a minority to the variably rectifying class; there were no in- or outwardly rectifying cells at all 34. This is a clear distinction from microglia 35 and oligodendrocytes 36 expressing exclusively inwardly rectifying voltage-current relationships.

Then, in the voltage-clamp recording mode of the amplifier, the holding potential of both astrocytes and neurons was set to -80 mV. Agonists and antagonists were applied locally, by means of a computer-controlled solenoid valve-driven pressurized superfusion system (VC^3^8; Ala Scientific Instruments, Farmingdale, NY, USA). The drug-application tip touched the surface of the brain slice and was placed 100-150 µm from the patched cell. The agonists NMDA (100 µM) and Bz-ATP (1000, 3000 µM) were superfused for 10 s both to astrocytes and neurons, spaced 3-min apart, and the current amplitudes caused by the two applications of Bz-ATP (1000 µM) were averaged.

Excitatory postsynaptic currents (EPSCs) were generated by placing a concentric platinum-iridium bipolar stimulating electrode (KD-CS, KedouBC, Suzhou, China) in the dendritic region of the CA1 neuron patched (100-150 µm distance from recording, near the outer border of the CA1 region). Two stimuli (7 mA strength, 100 µs duration each) with an inter-pulse interval of 50 ms were delivered every 20 s by a square wave stimulator (Master 9, MicroProbes for Life Science, Gaithersburg, MD, USA) and its coupled stimulus isolation unit (ISO-Flex, MicroProbes for Life Science). We stimulated with the above parameters for 5 min before, 10 min during the application of the P2X7R antagonist A438079 (10 µM), and again 5 min after washing out this drug with normal aCSF (Figure 9G-J). All EPSC amplitudes were measured at their peaks; the paired-pulse ratio (PPR) was calculated after dividing the second peak amplitude (P2) by the first peak amplitude (P1). The P2/P1 ratios were determined during the last 2 min before, during and after applying A438079. In some of the experiments we stimulated CA1 neurons only with a single pulse, but otherwise the conditions were similar to those described for paired-pulse stimulation; an important difference was, however, that low X^2+^ aCSF was used throughout.

Spontaneous postsynaptic currents (sPSCs) and spontaneous (s)EPSCs were recorded from neurons at the holding potential of -80 mV in a low X^2+^ aCSF. These signals were analyzed by means of the pClamp 10.4 software package, by detecting amplitudes exceeding the detection threshold set at three times the standard deviation above the baseline noise of the recordings 37. False positive noise-triggered fluctuations and signals with a non-monotonic rising phase and/or additional events within the decaying phase were rejected on visual inspection (< 2%). Since sPSCs compose of both action potential-induced and spontaneous vesicular glutamate/GABA release, the blockade of GABA_A_Rs by gabazine (10 µM) left us with pure glutamatergic currents. All electrophysiological experiments were performed on hippocampal slices prepared from mice injected 1 h before with saline or KA.

### Extracellular recordings

Hippocampal slices of 400 µm thickness were prepared for these experiments, 1 h after inducing KA-SE, otherwise as described above. Long-term potentiation (LTP) was recorded in the current-clamp mode of the amplifier (see below) in normal Ca^2+^-aCSF, with micropipettes filled with 1 M NaCl (2-5 MΩ tip resistance). The recording micropipette was positioned extracellularly in the *stratum radiatum* of the CA1 area targeting the distal dendrites of pyramidal neurons, and the concentric platinum-iridium bipolar stimulating electrode was placed near the CA3-CA1 border as shown in the inset of Figure 10A. Field excitatory postsynaptic potentials (fEPSPs) evoked by electrical stimulation with Master 9 and ISO-Flex (both from MicroProbes for Life Science) were recorded by means of a patch-clamp amplifier (MultiClamp 700B, Molecular Device). Square wave pulses of 100 µs duration were delivered every 20 s throughout. At first, input-output curves were generated for each slice in normal aCSF, and for subsequent experimentation, the stimulation intensity was adjusted to evoke 50% of the maximum response. Then, baseline values were recorded for 30 min, and afterwards LTP was induced by applying high frequency stimulation (100 Hz for 1 s, repeated two times at an interval of 20 s) and recorded for a further 1 h in normal aCSF, or one containing the drugs under investigation. All slopes were normalized to the average slope of the baseline.

### Drugs

Drugs used were the following: kainic acid hydrate (KA), A438079 hydrochloride hydrate (MedChem Express, Monmouth Junction, NJ, USA); N-methyl-D-aspartate (NMDA) (Merck, Shanghai, China); 2'(3')-*O*-(4-benzoylbenzoyl)adenosine-5'-triphosphate tri(triethylammonium) salt (Bz-ATP); istradefylline (KW6002), SCH58261 (AbMole Sci., Houston, TX, USA); JNJ-47965567, gabazine hydrobromide (Tocris Biosciences, Bristol, UK).

Kainic acid was dissolved in saline, while JNJ-47965567 was dissolved in 30% SBE-β-CD + 70% saline. SCH58261 and KW6002 were dissolved in 15% DMSO + 85% saline. The stock solutions of Bz-ATP and NMDA were dissolved in distilled water, while that of A438079 in 10% DMSO; these solutions were further diluted in aCSF.

### Data analysis

All data were expressed as means ± SEM of *n* observations, where *n* means the number of animals in all experiments, with the exception of the electrophysiological measurements, where it means the number of experiments from at least 3 animals. GraphPad Prism10 was used for the construction of Figures and for statistical evaluations. We tested whether the sampled distribution of data satisfied the normality and equal variance criteria with the Kolmogorov-Smirnov test. Multiple comparisons between data with one variable were performed by one-way ANOVA, while multiple comparisons between data were performed in case of their non-normal distribution, by the Kruskal-Wallis ANOVA on ranks. Multiple comparisons of data with two variables were performed with two-way ANOVA. As a *post hoc* test, the Tukey's multiple comparison test was used. Comparison of two data sets were made by the parametric, unpaired Student's t-test or the non-parametric Mann-Whitney test. A probability level of 0.05 or less was considered to be statistically significant.

## Results

### Seizures evoked by kainic acid injection

In the first series of experiments, we intended to find out whether the i.p. injection of KA (30 mg/kg) to mice causes an SE corresponding to seizure stage 4-5 in the Racine scale (Figure 1). Within the 60 min observation time, a gradual increase of seizure intensity developed, reaching a maximum in the last 20 min. We also tested, whether the i.p. injection of the blood-brain barrier (BBB) permeable P2X7R antagonist JNJ-47965567 (30 mg/kg) 1 h before KA application interfered with seizure activity (Figure 1A). We found that this was the case, in that in JNJ-47965567 co-treated mice, between 30-40 min and 40-50 min after KA injection the mean seizure stage was significantly lower than without this co-treatment. Correspondingly, the mean onset until SE was lengthened, and the mean duration of SE was decreased, when JNJ-47965567 and KA were sequentially applied (Figure 1B, C). The BBB permeable A2AR antagonists KW6001 and SCH58261 (30 mg/kg, i.p. each) had the same effects, both on the onset of SE (Figure 1E, H) and the duration of SE (Figure 1F, I), although the inhibition of the respective seizure stage *vs.* time curves did not reach the threshold of statistical significance (Figure 1D, G). There were no seizures observed 24 h after SE.

### Experimental design and proof of cell-specific labeling by a genetic Ca^2+^ indicator

These findings allowed us to design the time-course of all subsequent experiments (Figure S1A). Our next aim was to measure intracellular Ca^2+^ dynamics in neurons and astrocytes of the CA1 region of the hippocampus, as both cell types are known to be involved in the steering of SE 22. For this purpose we micro-injected the hippocampal CA1 region either with the neuron-specific (rAAV-hSyn-GCaMP6f-EGFP) or astrocyte specific (rAAV-GfaABC1D-GCaMP6f-EGFP) virus-complexes initiating the cell-specific labeling with the genetic Ca^2+^ indicator after a delay of 2 weeks (see Methods; Figure S1B-E). Thus, 2 weeks after microinjection of the viruses to the hippocampus, we i.p injected KA (30 mg/kg) or an equivalent amount of solvent, with or without a preceding i.p. injection of a P2X7R (JNJ-47965567; 30 mg/kg) or A2AR antagonist (KW6002 or SCH58261; 3 mg/kg, each). The fiber photometric measurement of Ca^2+^ signals (or in separate experiments the determination of ATP/adenosine release or the preparation of hippocampal brain slices for electrophysiology) was carried out by keeping an interval of 1 h after KA application. About 24 h later, these measurements were repeated, and behavioral and electrophysiological investigations took place as well.

Immunohistochemistry confirmed that green fluorescence protein (EGFP), labelling the presence of rAAV-hSyn-Cyto-GCaMP6f-EGFP in neurons, and red fluorescence of the astrocytic marker GFAP, produced no overlay, while the same EGFP co-stained with the neuronal marker NeuN (Figure S1B, C). By contrast, green fluorescence, labelling the presence of rAAV-GfaABC1D-GCaMP6f-EGFP (astrocyte-specific), and red fluorescence, labelling the presence of the astrocytic marker GFAP, clearly co-stained, while there was no co-staining of EGFP with the neuronal marker NeuN. We were not able to quantify these experiments, because the neurons and astrocytes in the CA1 region appear in high densities and could not be well discerned from each other in the specimens. Nonetheless, the specificity of the expression of a genetically encoded Ca^2+^ indicator was well documented by the examples chosen out of 4 similar images.

### Ca^2+^ dynamics in neurons and astrocytes of the hippocampal CA1 area and the medial prefrontal cortex after kainic acid-induced status epilepticus

Two weeks after the injection of a virus-complex to label cells with the genetic Ca^2+^ indicator, we measured by fiber photometry the changes in Ca^2+^ signals in CA1 neurons, 1 h after i.p. KA (30 mg/kg) injection, in comparison with the corresponding changes after an equivalent quantity of saline injection (Figure 2A, left panel). We also recorded the EEG signals after KA from the same mice, and compared them with the EEG measured after saline injection (Figure 2B, left panel). The ΔF/F_0_ value and the EEG signals were both expressed in the respective heatmaps (Figure 2A, B, middle panels) and also documented as the AUC of saline and KA (Figure 2A, right panel) as well as the total power (n-fold change) of saline and KA (Figure 2A, right panel). Hence, 1 h after KA injection a large increase of both the Ca^2+^ signal AUC and the EEG total power developed.

24 h after KA injection there was no change in the AUC in comparison with that of saline-treated animals (Figure 2C, right panel) and a very small difference between the total power (n-fold) of the KA animals in comparison with the saline-treated ones (Figure 2D, right panel). It is important to mention, that the maximum values, and in consequence, the scaling at the Y-axes show much smaller AUC and total power values in Figure 2C than in A, and in Figure 2D than in B, although the absolute length of the Y-axes is the same.

In view of the findings described above which show that the P2X7R antagonist JNJ-47965567 (30 mg/kg, i.p.) delayed the onset and decreased the duration of KA (30 mg/kg)-induced behavioral SE, we investigated the effects of the sequential application of these two substances on the hippocampal CA1 Ca^2+^ signals and EEG activity. We noted that JNJ-47965567 inhibited the AUC of the Ca^2+^ signal, in comparison with that obtained by the injection of saline (Figure 2E) and had a similar effect on the EEG total power (Figure 2F). 24 h after KA injection, the differences in KA-induced AUC and EEG power from the respective saline-induced activities have shown no or only negligible changes (Figure 2G, H).

While in the above experiments we concentrated on CA1 neurons, subsequently we turned our attention to CA1 astrocytes. We again used fiber photometry to observe the ΔF/F_0_ changes in astrocytes selectively labeled with Ca^2+^ indicator in response to i.p. KA, in comparison with the corresponding changes after saline injection (Figure 3A). The effect of KA on Ca^2+^ signal in CA1 neurons (increase to 550.9 ± 52.7% of the control) was similar as in CA1 astrocytes (increase to 462.7±53.6%, n = 5 each; unpaired t-test, t = 1.173, P = 0.2745). On the same mice we also recorded the EEG after KA and saline injection (Figure 3B, left panel).

In agreement with findings on CA1 neurons, CA1 astrocytes produced a marked increase in Ca^2+^ signaling in a JNJ-47965567-sensitive manner (compare Figure 3A with Figure 3E). The response to KA was time-dependent; it was quite pronounced 1 h after KA injection, but was practically absent on the following day (compare Figure 3A with Figure 3C). The composition of Figure 3 is similar to that of Figure 2; it documents the mean ΔF/F_0_ effects, with their heatmaps, and AUC values for each treatment condition. In addition, it also displays the EEG recordings, their heatmaps, and the total power values, again for each treatment condition. Hence, the quite modest, residual Ca^2+^ signal-AUC and EEG power determined in the presence of JNJ47965567 was almost non-existent 24 h after KA-injection (Figure 3H).

**Figure 4:**
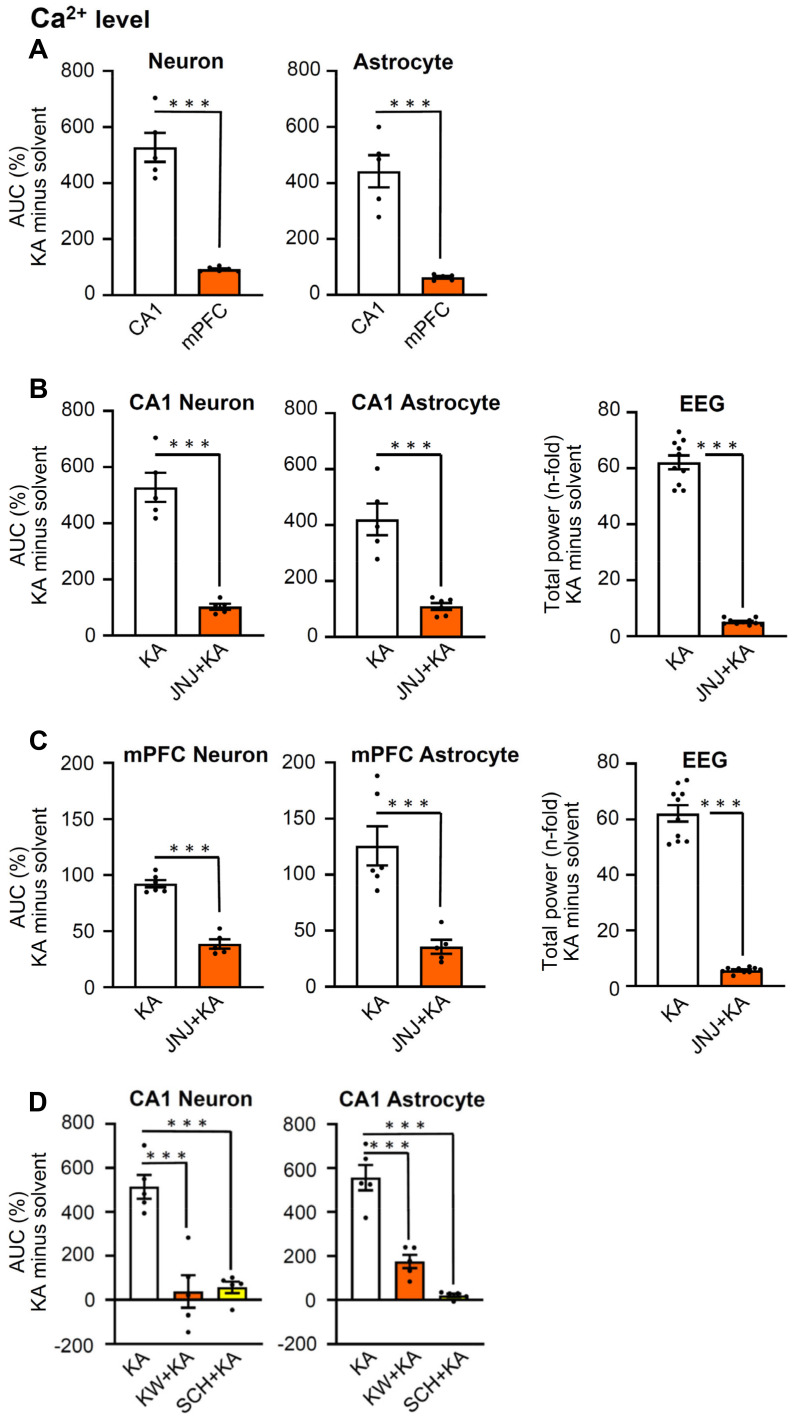
Effects of kainic acid (KA; 30 mg/kg, i.p.) injection on Ca^2+^ signals in neurons and astrocytes in the CA1 area of the hippocampus and in the medial prefrontal cortex (mPFC). In some of the experiments, the n-time change of the EEG total power was also measured. The increase of Ca^2+^ signals was prevented by the P2X7R antagonist JNJ-47965567 (30 mg/kg, i.p.), as well as by the A2AR antagonists KW6002 and SCH58261 (3 mg/kg, i.p., each). The time-course of the experiments and further methodological details are described in the Legends to Figures 1 and 2. In contrast to these Figures, the effect of KA is presented as AUC minus the solvent-induced effect. (**A**) The increase of Ca^2+^ signals by KA was similar in neurons and astrocytes of the hippocampal CA1 region and the mPFC each, but this increase was much smaller in the latter than in the former. (**B**) JNJ-47965567 greatly inhibited the KA-induced increases in the Ca^2+^ concentration both in neurons and astrocytes of the CA1 area of the hippocampus. Similarly, JNJ-47965567 markedly inhibited the EEG activity. (**C**) JNJ-47965567 greatly inhibited the KA-induced increases in the Ca^2+^ concentration both in neurons and astrocytes of the mPFC. JNJ-47965567 again markedly inhibited the EEG activity. (**D**) KW6002 and SCH58261 also strongly inhibited the Ca^2+^ concentration increase in CA1 neurons and astrocytes. The levels of statistical significance reached are marked with *P < 0.05, **P < 0.01, and ***P < 0.001. The unpaired t-test was used for evaluation in **A-C** (**a, left**, t = 9.270; **A, right**, t = 6.528; **B, left**, t = 8024, **B, middle**, t = 5.893; **B, right**, t = 22.91; **C, left**, t = 10.23, **C, right**, t = 19.11). The number of experiments was 5 for CA1 neurons and astrocytes, as well as 6 for mPFC neurons and astrocytes. The number of experiments was 10 for the EEG measurements. The two-way ANOVA was used for evaluation in **D, left** (Row F_4,8_ = 3.883, P = 0.0486; Column F_2,8_ = 47.11, P < 0.0001; KA vs. KW+KA, P < 0.0001; KA vs. SCH+KA, P < 0.0001) and **D, right** (Row F_4,8_ = 0.5372, P = 0.7131; Column F_2,8_ = 45.18, P < 0.001; KA vs. KW+KA, P < 0.0004; KA vs. SCH+KA, P < 0.0001).

Since hippocampal CA1 neurons have multiple functions, they, for example, steer epileptic seizures, but in addition also contribute to depressive-like behaviors, the latter in conjunction with the medial prefrontal cortex (mPFC; 21,38); therefore, we decided to make similar experiments as reported above, also in the mPFC. We chose to plot the effects of KA on neuronal and astrocytic Ca^2+^ signaling (ΔF/F_0_ changes expressed as AUC values), and the EEG changes expressed as total power values. For this purpose, the solvent data were deduced from the KA data. Figure 4A shows that the KA-induced AUC minus solvent values of neurons in CA1 neurons and astrocytes were much larger than the corresponding changes in mPFC neurons and astrocytes. Otherwise, JNJ-47965567 similarly inhibited the AUC minus solvent values both in neurons and astrocytes, in the CA1 and mPFC areas (Figures 4B, C, left and middle panels). The EEG total power and its inhibition by JNJ-47965567 for KA minus solvent results was also identical in the CA1 and mPFC areas (Figure 4B, C, left panels). Likewise, the KA-induced AUC values for Ca^2+^ signaling in CA1 neurons and astrocytes, were equally inhibited by the A2AR antagonists KW6002 and SCH58261, when injected i.p. in doses of 3 mg/kg, each (Figure 4D). The original experiments which served to generate the data for the mPFC in Figure 4, are shown in Figures S2 and S3.

### Release of ATP and adenosine in the CA1 area of the hippocampus after kainic acid-induced *status epilepticus*

**Figure 5:**
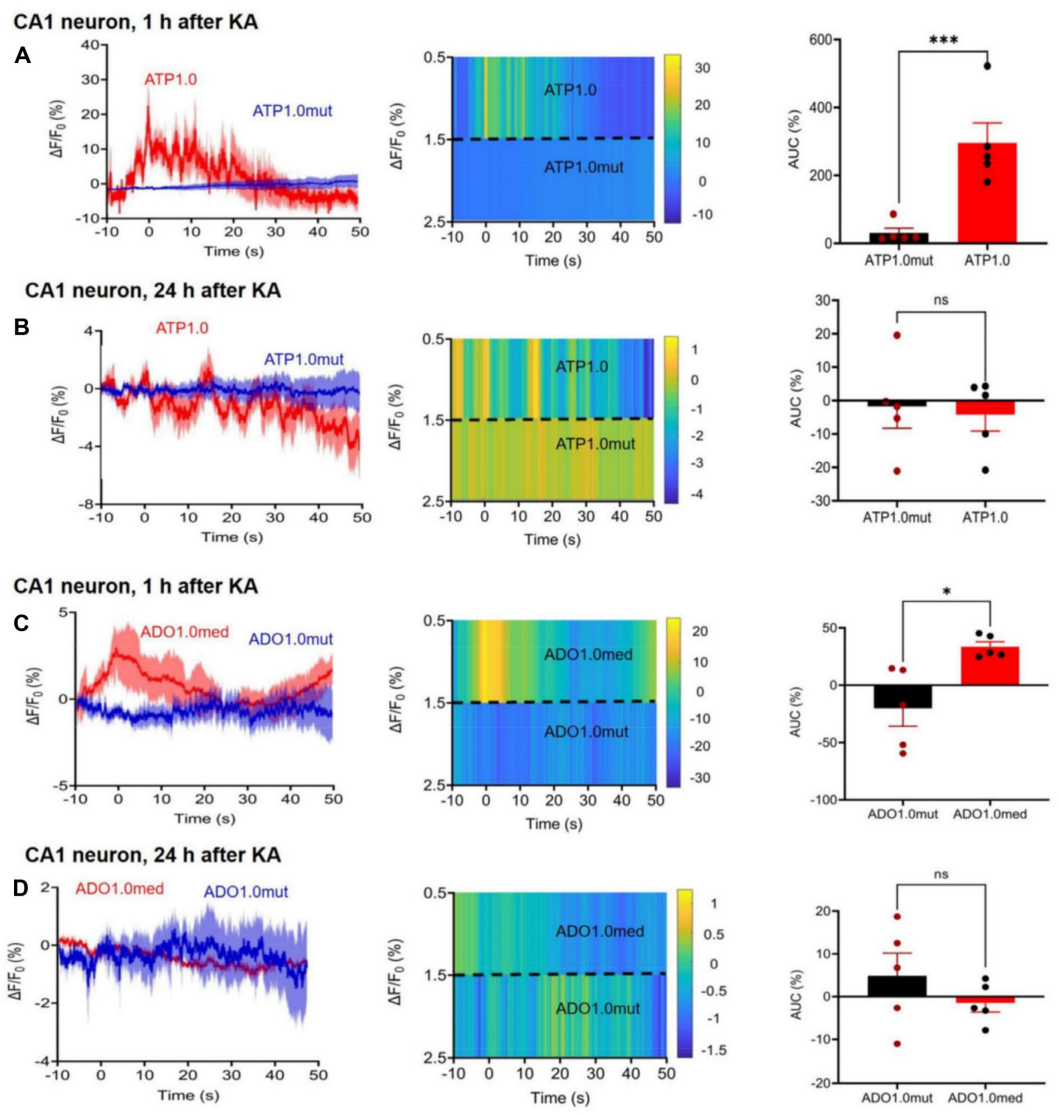
Effect of KA injection (30 mg/kg, i.p.), 2 weeks after microinjection into the hippocampal CA1 area rAAV-hSyn-ATP1.0 to initiate the synthesis of a neuron-specific ATP-responsive sensor or its control virus, rAAV-hSyn-ATP1.0mut; in other experiments rAVV-hSynAdo1.0med was microinjected into the CA1 area to induce the synthesis of an adenosine (ADO)-responsive sensor or its control virus, rAVV-hSyn-Ado1.0mut. (**A**, **left**) The ΔF/F_0_ fluorescence ratio obtained was a measure of the ATP release, 1 h after KA injection, in the CA1 area around neurons. The heatmap qualitatively characterizes the time-dependent release of ATP (**A, middle**), while the area under the curve (AUC) gives a quantitative measure of the released ATP (**A, right**). (**B**) Analogous presentation of ATP release 24 h after KA injection. Analogous presentation of adenosine release around CA1 neurons, 1 h (**C**) and 24 h (**D**) after KA injection. The composition of the panels was the same as that in **A, B**. The levels of statistical significance reached are marked with *P < 0.05, **P < 0.01, and ***P < 0.001, n.s. P > 0.05. The unpaired t-test was used for evaluation throughout (**A**, t = 10.00; **B**, t = 0.2969; **C**, t = 3.306; **D**, t = 1.106). The number of experiments was 5 in each panel. The release of ATP was much larger than the release of adenosine (AUC **A** vs. AUC **C**; t = 4.406, P = 0.0023).

Since both P2X7R and A2AR antagonists appeared to interfere with the KA-induced neuronal and astrocytic Ca^2+^ signaling increases in the CA1 area of the hippocampus, the next logical step was to answer the question, whether ATP and adenosine, the endogenous ligands of these receptors are released by KA injection around CA1 neurons and astrocytes (Figure 5). Fiber photometry offers an adequate tool to answer this question. The GRAB-sensor rAAV-hSyn-ATP1.0 and its negative control rAAV-hSyn-ATP1.0-mut allowed reliable and time-dependent measurements of ATP release in the CA1 area of the mouse hippocampus, 2 weeks after their micro-injection (Figure 5A, B). Similarly, rAAV-hSyn-Ado1.0 and its control rAAV-hSyn-Ado1.0mut could be used for determining the adenosine release at the same region of the brain (Figure 5C, D). KA injection caused after 1 h a rapidly increasing and somewhat slower declining release of ATP, and the release of adenosine had a similar time-course. 24 h after KA injection there was no comparable release of ATP/adenosine observed.

### Impairment of spatial- and non-spatial hippocampus-dependent cognitive abilities, and no change of general behavior in the open-field apparatus, after kainic-acid-induced *status epilepticus*

**Figure 6:**
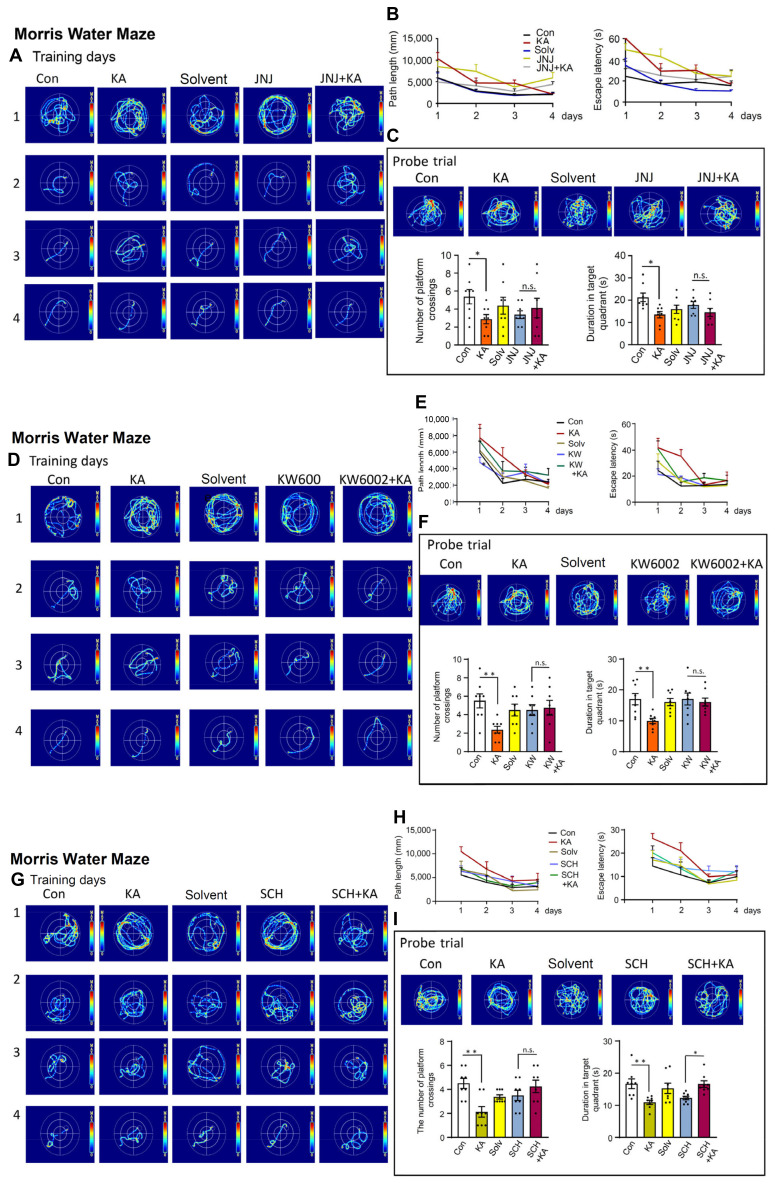
Effects of kainic acid (KA; 30 mg/kg, i.p.) on hippocampus-dependent spatial memory of mice in the Morris Water Maze test, when applied alone or in combination with JNJ-47965567 (30 mg/kg, i.p.), KW6002, or SCH58261 (3 mg/kg, i.p., each). Four training days were followed by a probe trial on the 5^th^ day, as described in the Methods Section. The tracks covered by the mice during the training days are shown in **A, D, G**. After the last training day measurement, JNJ-47965567 or solvent injection was followed after a 1 h-interval by KA or its solvent application (**A**). Then, the next day the probe trial measurement was carried out. The results of the path length and escape latencies on each day are shown in **B**. The number of platform crossings and duration of stays in the target quadrant are presented in **C**. KA was dissolved in saline, while JNJ-47965567 was dissolved in 30% SBE-β-CD + 70% saline. With an analogous application protocol, KW6002 (**D-F**) or its solvent, or SCH58261 or its solvent (**G-I**; 15% DMSO + 85% saline, in each case) were injected i.p. Con, control; Solv, solvent. Two-way ANOVA was used in this (**C**) and also in the next sets of experiments (**F, I**) for statistical evaluation; the levels of significance reached are marked with *P < 0.05, **P < 0.01, ***P < 0.001, n.s. P > 0.05 (**C below left,** Row F_7,28_ = 0.4175, P = 0.8832; Column F_4-28_ = 2.305, P = 0.0292; **C below right**, Row F_7-28_ = 0.5680, P = 0.7754, Column F_4,28_ = 2.953, P = 0.0374; **F below left,** Row F_7-28_ = 1.027, P = 0.4345, Column F_4-28_ = 3.309, P = 0.0243; **F below right,** Row F_7-28_ = 0.9130, P = 0.5110, Column F_4-28_ = 3.957, P = 0.0114; **I below left,** Row F_7-28_ = 0.9806, P = 0.4647, Column F_4-28_ = 4.995, P = 0.0036; **I below right,** Row F_7-28_ = 2.1185, P = 0.0668, Column F_4-28_ = 6.348, P = 0.0009). The number of experiments was 8 in each column.

After having documented the increase of CA1 neuronal and astrocytic [Ca^2+^]_i_ by KA-induced SE, we decided to find out whether CA1-dependent spatial learning is impaired by SE, and can be reversed by P2X7R and A2AR antagonism. For this purpose we made use of the MWM system, routinely employed to measure hippocampus-dependent spatial learning processes. The i.p. injection of KA (30 mg/kg) inevitably led to a suppressed cognitive performance of mice after 4 days of training on the 5^th^ day in a probe trial (Figure 6A-I). 24 h before the start of the probe trial measurement we injected either KA (30 mg/kg, i.p.) or an equivalent quantity of solvent. The injection of JNJ-47965567 (30 mg/kg, i.p.) preceding the injection of KA, counteracted the cognitive deterioration, when evaluated in comparison with the injection of JNJ-47965567 alone (Figure 6C). These changes were evident both with respect to the numbers of platform crossings and the duration of detentions in target quadrants. JNJ-47965567 alone had no effect on any of these parameters, when compared with the effect of the solvent (Figure 6C). The path length and the escape latency during the training days is also depicted, but was not statistically evaluated, although there was apparently a continuous improvement of the spatial learning ability (Figure 6B). The images of the mouse running tracks on the training days and the day of the probe trial are presented in Figure 6A, C.

Because P2X7- and A2AR-stimulation was shown to equally relieve the behavioral seizures as well as the hippocampal Ca^2+^ responses, we asked ourselves, whether the activation of these receptors might also counteract the cognitive limitation observed in the MWM. Indeed the two A2AR antagonists KW6002 and SCH58261 (3 mg/kg, i.p., each), equally and in a similar manner as JNJ-47965567, reversed the deleterious effect of KA on the cognitive abilities measured on the day of the probe trial (Figure 6F and I).

**Figure 7:**
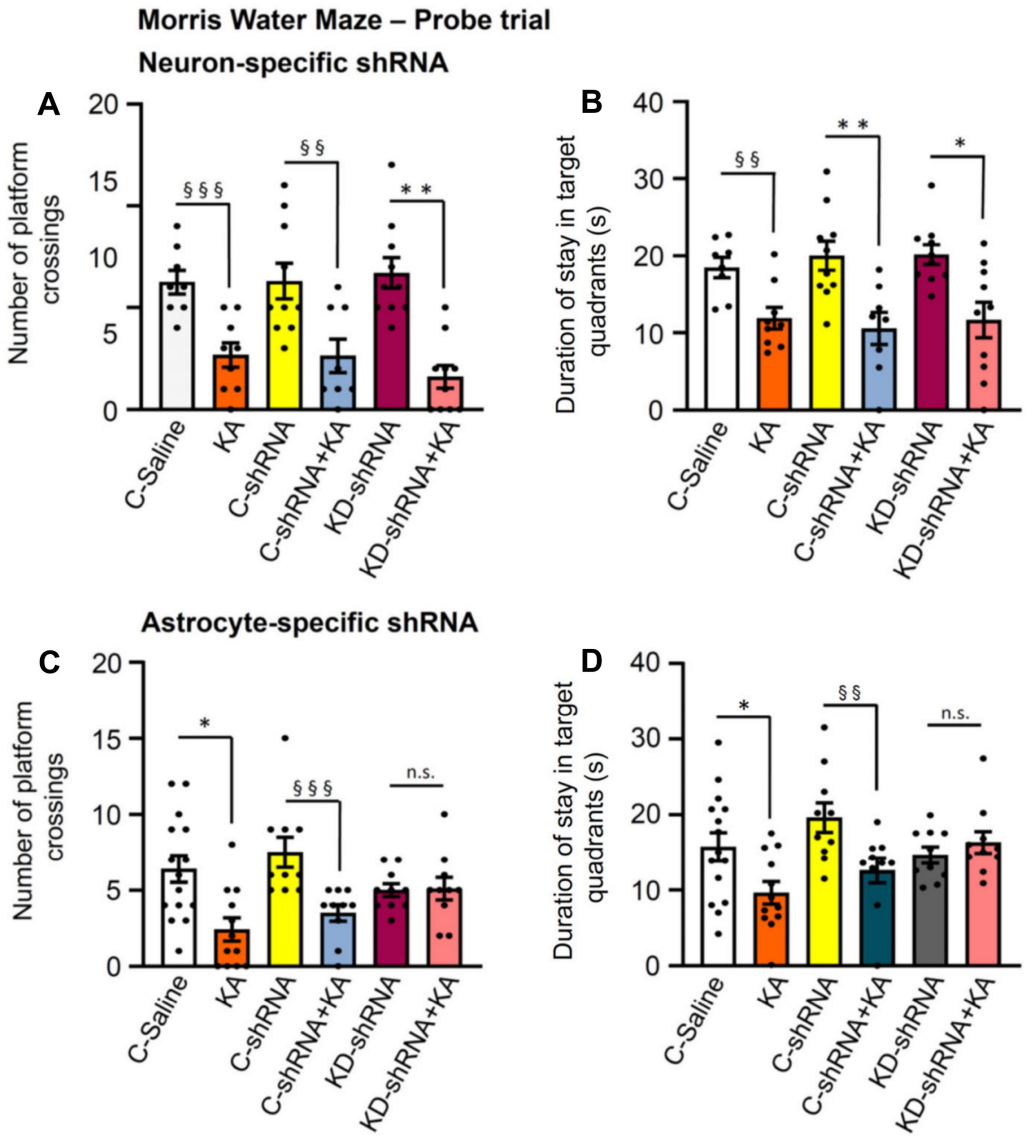
Probe trial data of the Morris Water Maze test in control mice or mice injected with kainic acid (KA; 30 mg/kg, i.p.) after previous micro-injection into their lateral ventricles of neuron-specific or astrocyte-specific shRNAs directed against P2X7Rs. KA was dissolved in saline and was injected 24 h before the collection of data on the day of the probe trial. A group of mice was injected with P2X7R shRNA and another group with control shRNA lacking mP2X7, which is responsible for the knockdown of the receptor. The results show that the neuron-specific abrogation of P2X7Rs does not change the cognitive achievements of mice in the MWM test, whereas the astrocyte-specific abrogation of this receptor inhibits the cognitive achievements. C, control; KD, knockdown; MWM, Morris Water Maze. Effect of neuron-specific shRNA on the number of platform crossings (**A**) and the duration of stays in target quadrants (**B**) (mean ± SEM of 8-10 mice). Kruskal-Wallis ANOVA on ranks (**A**) or one-way ANOVA (**B**) was used for statistical evaluation; the levels of significance reached are marked with *P < 0.05, **P < 0.01 (**A**, Groups = 6, Kruskal-Wallis statistics = 29.27, P < 0.0001; Dunn's test, KD-shRNA vs. KD-shRNA+KA, P = 0.0011; **B**, F_5,49_ = 6.568, P < 0.0001; Dunn's-test, C-shRNA vs. C-shRNA+KA, P = 0069, KD-shRNA vs. KD-shRNA+KA, P = 0.0110). In addition, pairs of columns were compared with each other by the unpaired t-test or the Mann-Whitney test, as adequate and the levels of significance are marked with ^§§^P < 0.01, ^§§§^P < 0.001. **A** (C-Saline vs. KA, t = 4.234; C-shRNA vs. C-shRNA+KA, t = 2.982; KD-shRNA vs. KD-shRNA+KA, t = 5.393) and **B** (C-Saline vs. KA, t = 3.414). Effect of astrocyte-specific shRNA on the number of platform crossings (**C**) and the duration of stays in target quadrants (**D**) (mean ± SEM of 10-15 mice). Kruskal-Wallis ANOVA on ranks (**C, D**) was used for statistical evaluation; the levels of significance reached are marked with *P < 0.05, n.s. P > 0.05 (**C**, Groups = 6, Kruskal-Wallis statistics = 21.62, P = 0.0002; Dunn's test, C-Saline vs. KA, P = 0.0222; **D**, Groups = 6, Kruskal-Wallis statistics = 15.25, P = 0.0093). In addition, pairs of columns were compared with each other by the unpaired t-test or the Mann-Whitney test, as adequate and the levels of significance are marked with ^§§^P < 0.01, ^§§§^P < 0.001, n.s. P > 0.05 (**C**, C-shRNA vs. C-shRNA+KA, Mann-Whitney U = 4.5; **D**, C-shRNA vs. C-shRNA+KA, Mann-Whitney U = 17).

Recently a major dispute emerged on the question, whether P2X7Rs are present in neurons, or whether their effects develop indirectly due to the release of glial signaling molecules/metabolic byproducts 39,40. Our own group favored the idea that P2X7Rs are preferentially or even exclusively located at astrocytes instead of neurons. In order to decide this question with respect to the KA-SE-induced impairment of spatial memory, we micro-injected to the CA1 area of the hippocampus of mice shRNA, which cell-specifically abrogated P2X7Rs in neurons or astrocytes (Figure 7A-D). For the sake of simplicity, we evaluated only the probe trial results of the MWM, in mice which obtained neuron-specific, or astrocyte specific shRNA in their lateral cerebral ventricles, and compared these results with those generated in mice which obtained the respective control shRNA. In accordance with previous experiments, KA (30 mg/kg s.c., injected 1 d before the probe trial), caused an impairment of both the number of platform crossings and the duration of stays in the target quadrants. The microinjection of neuron-specific shRNA two weeks before measurement did not interfere with the cognitive deterioration (Figure 7A, B), whereas the microinjection of astrocyte-specific shRNA abolished it (Figure 7C, D). Hence, we conclude that astrocytes rather than neurons are involved in the P2X7R-mediated depression of cognitive abilities.

**Figure 8:**
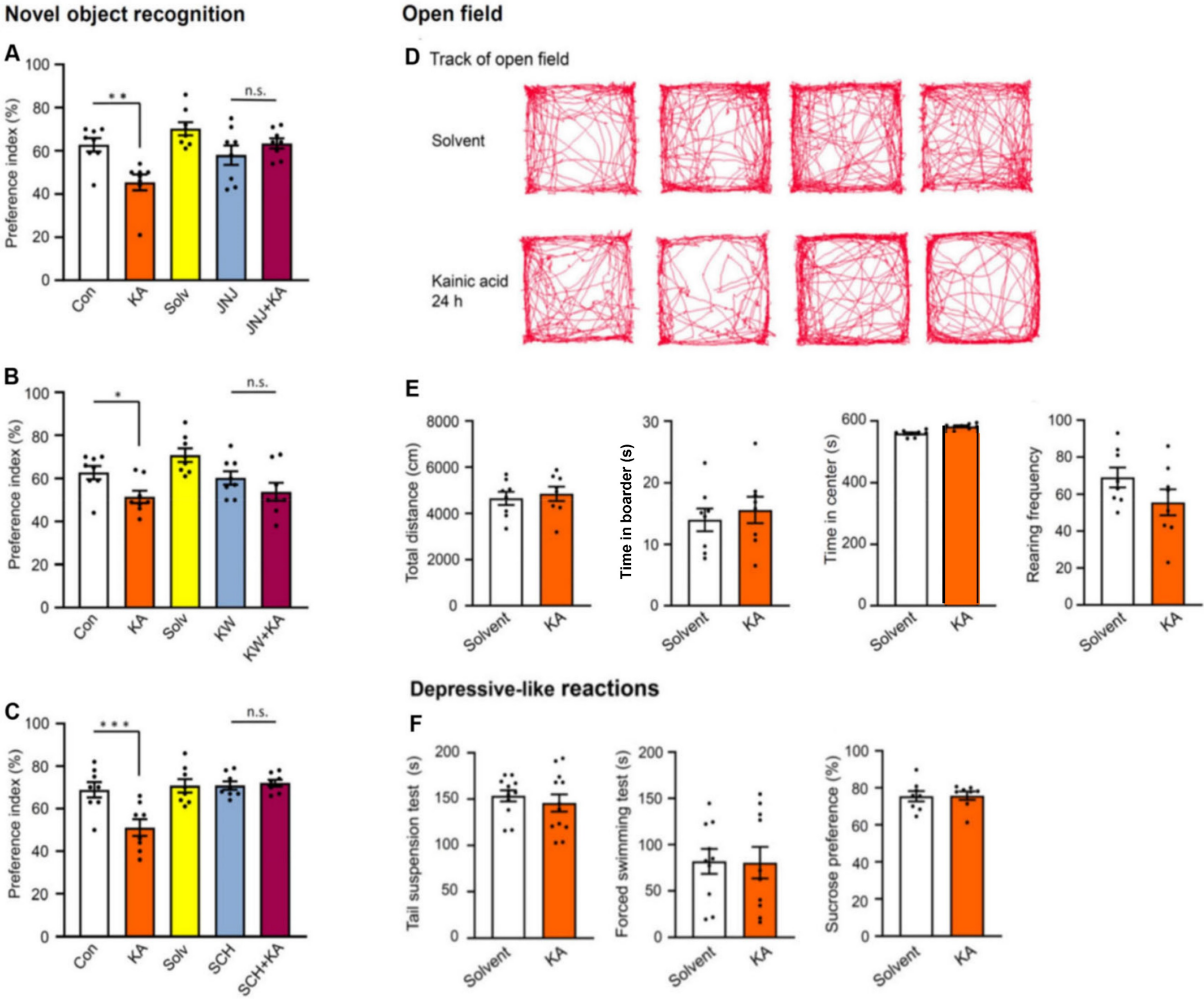
Effects of kainic acid (KA; 30 mg/kg, i.p.) on hippocampal-dependent non-spatial memory of mice in the Novel Object Recognition test, when applied alone or in combination with JNJ-47965567 (30 mg/kg, i.p.), KW6002, or SCH58261 (3 mg/kg, i.p., each) (**A-C**). On the first 2 days mice were trained to recognize an object as described in the Methods Section. Then, KA was injected, and on the following day the time spent with the novel object and the preference index for the correctly recognized objects were determined. Further details are described in the Methods Section. KA was dissolved in saline, while JNJ-47965567 was dissolved in 30% SBE-β-CD + 70% saline. With an analogous application protocol, JNJ-47965567 or its solvent (**A**), KW6002 or its solvent (**B**; 15% DMSO+85% saline), or SCH58261 or its solvent (**C**; 15% DMSO + 85% saline) were injected i.p. Con, control; Solv, solvent. Two-way ANOVA was used in all experiments for statistical evaluation; the levels of significance reached are marked with *P < 0.05, **P < 0.01, ***P < 0.001, n.s. P > 0.05 (**A left,** Row F_7,28_ = 1.257, P = 0.3066; Column F_4-28_ = 14.02, P < 0.0001; **A right,** Row F_7,28_ = 1.876, P = 0.1118; Column F_4-28_ = 8.668, P < 0.0001; **B left,** Row F_7,28_ = 1.286, P = 0.2930; Column F_4-28_ = 19.37, P < 0.0001; **B right,** Row F_7,28_ = 0.7618, P = 0.6234; Column F_4-28_ = 6.513, P < 0.0008; **C left,** Row F_7,28_ = 1.729, P = 0.1426; Column F_4-28_ = 12.52, P < 0.0001; **C right,** Row F_7,28_ = 0.9046, P = 0.5170; Column F_4-28_ = 8.596, P < 0.0001). The number of experiments was 8 in each column. Effects of kainic acid (KA; 30 mg/kg, i.p.) and its solvent in the open field test (**D, E**). The tracks of mice, when injected with KA or its solvent (**D**). Effects of KA and its solvent on the total running distance, the time spent in the center, the time spent in the border and the rearing frequency (**E**). None of these parameters changed in comparison with their solvent treated counterparts. The unpaired t-test was used to check statistically significant differences (**E left,** t = 0.4654; **E left middle,** t = 0.5673; **E right middle,** t = 0.7624; **E right,** t = 1.521). The number of experiments was 8 in each column. The acute depressive-like reactions in the tail suspension test (TST), forced swim test (FST) and sucrose consumption test (SCT) also did not differ in comparison between solvent- and KA-induced effects. The unpaired t-test was used to check statistically significant differences ( **F left,** t = 0.7031; **F middle,** t = 0.6666; **F right,** t = 0.8979). The number of experiments was 10 (TST) and 8 (FST, SCT).

Then, a model of non-spatial memory, the Novel Object Recognition test (NORT), was applied to mice, 24 h after KA application, in order to examine whether a general memory deficit or a memory deficit only limited to spatial orientation is affected by SE. Similar to MWM, NORT also probes hippocampal-dependent memory. KA injection markedly decreased the time spent with the novel object (preference index; Figure 8A-C). JNJ-47965567 (30 mg/kg, i.p.), KW6002, and SCH58261 (3 mg/kg, i.p., each), were all applied in the previously used doses. All three antagonists prevented the inhibition of cognitive performance (Figure 8A-C), and in the case of JNJ-47965567, even an overshooting increase of the time spent with novel objects was observed (Figure 8A). The general conclusion derived from the MWM and NORT measurements is, that P2X7R and A2AR activation by the hippocampal release of ATP and adenosine, respectively, both subsequent to KA injection, equally worsened spatial and non-spatial memory.

After collecting this information, we set out to look for more general behavioral effects in the open-field apparatus (Figure 8D, E). The tracks of five individual mice during the 10 min observation time after solvent or KA-injection are shown in Figure 8D, and their statistical evaluation is presented below in Figure 8E. KA had no effect on the total running distance, time spent in the center, time spent in the border, and the rearing frequency of mice, when measured 24 h after its injection. Already the failure of KA to increase the duration of the time spent in the center argues against an anxiogenic/depressive-like effect, because normally mice are afraid of open spaces and prefer to stay at the border of the apparatus.

### P2X7 receptor-dependent fraction of the kainic acid-induced increase in the number of adult neural progenitor cells in the hippocampal subgranular zone of the dentate gyrus

In the following experiments, we counted the number of doublecortin (DCX)-immunopositive adult neural progenitor cells (NPCs) in the subgranular zone of the hippocampal dentate gyrus (Figure S4). KA-induced SE greatly increased the DCX^+^ cell number both in wild-type (WT) and P2X7R KO mice, 3 weeks after initiation of the seizures (Figure S4). However, whereas this cell number in the WT and P2X7R KO subgranular zones was similar, when preparations were taken from KA-untreated mice, it differed in the KA-treated ones. Thus, the genetic deletion of P2X7Rs appeared to relieve the moderate brake imposed on the KA-induced proliferation of DCX-immunopositive NPCs by this receptor.

### No change in acute stress-induced depressive-like reactions after kainic acid-induced *status epilepticus*

In view of the increase of Ca^2+^ signals in the mPFC (Figure 4C, left and middle panels) after KA injection, it was an obvious question to raise, whether the function of this depression-relevant area may be disturbed following SE. Therefore, we measured in three standard test systems, used to model learned helplessness/depressive-like behavior in mice, under acute conditions, the effects of KA injection (again 24 h after injection). The TST, FST, and SCT revealed no difference between the effects of solvent and KA (Figure 8F). Thus, there was no indication for depressive-like behaviors after SE.

### Electrophysiological measurement of pre- and postsynaptic effects of kainic acid-induced *status epilepticus* in hippocampal brain slices; agonist-induced currents and paired-pulse potentiation

**Figure 9:**
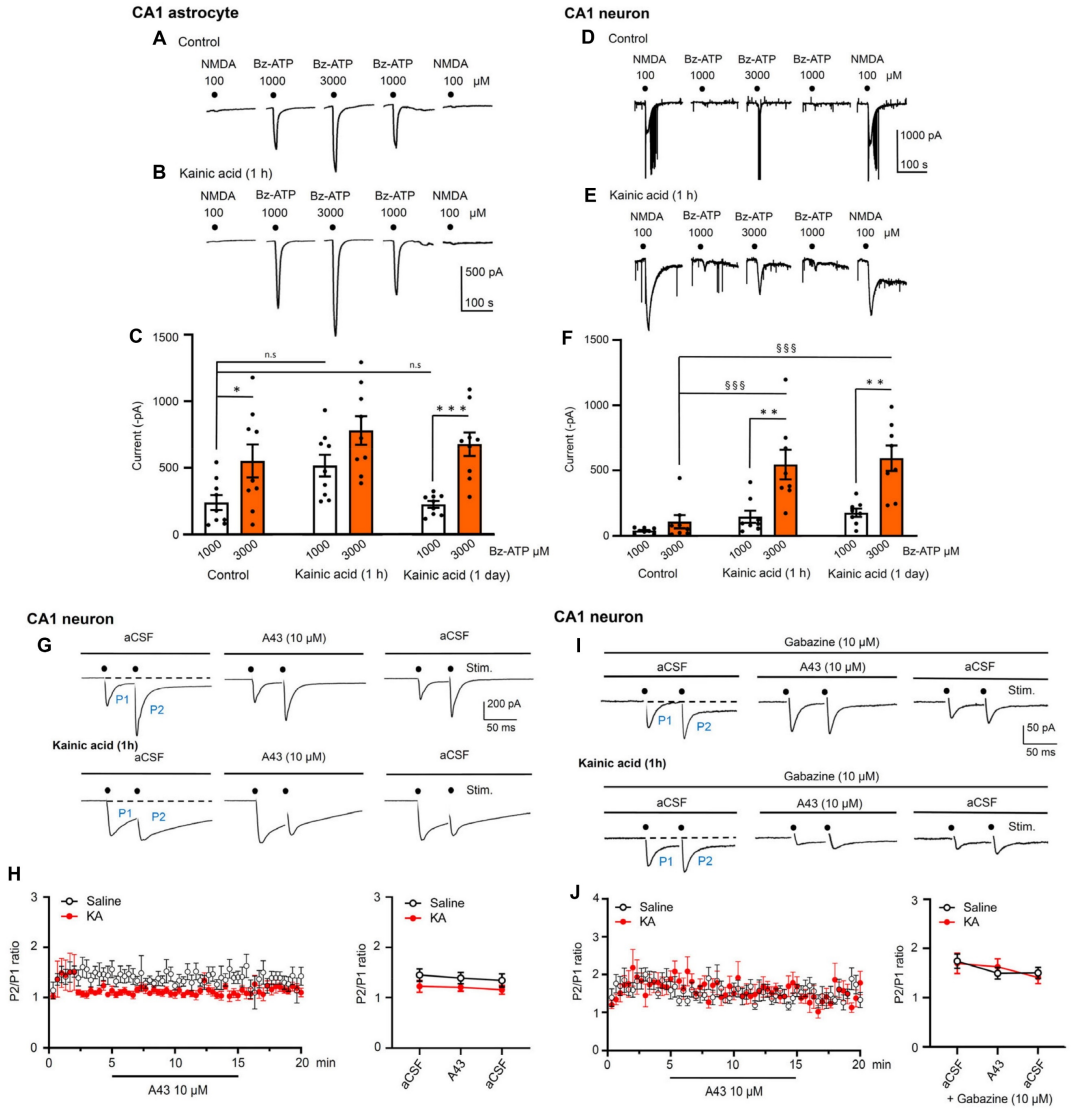
Effects of kainic acid (KA; 30 mg/kg, i.p.) on NMDA and Bz-ATP-induced current amplitudes in hippocampal CA1 neurons and astrocytes, 1 hour and 1 day after KA application; exclusion of a presynaptic modulation of glutamate release from CA1 neurons induced by double-pulse stimulation, with endogenously released ATP. Representative patch-clamp recordings of current responses to NMDA (100 µM) and Bz-ATP (1000, 3000 µM) from astrocytes and neurons without and with the preceding injection of KA (**A, B, D, E**). The mean ± S.E.M. current amplitudes for astrocytes (**C**) and neurons (**F**) are plotted in the respective panels. Further details are described in the Methods Section. One-way ANOVA was used for statistical evaluation; the levels of significance reached are marked with ^§§§^P < 0.001, n.s., P > 0.05 (**C,** F_5,48_ = 6.816, P < 0.0001; differences from control Bz-ATP 1000, P = 0.2301 and P > 0.9999, respectively; **F,** F_5,42_ = 12.06, P < 0.0001, differences from control Bz-ATP 3000, P = 0.0007 and P = 0.0001, respectively). The differences between the pairs of white and red columns were calculated by the t-test; the levels of significance reached are marked with *P < 0.05, **P < 0.01, ***P < 0.001 (**C,** control, t = 2.293, P = 0.0357; KA 1 h, t = 1.959, P = 0.0677; KA 1 d, t = 4.923, P = 0.0002; **F,** control, t = 0.2017, P = 1.340; KA 1h, t = 3.238, P = 0.0060; KA 1 d, t = 0.4075, P = 0.0011. Paired-pulse potentiation (PPP) in CA1 hippocampal neurons of saline- and KA (30 mg/kg, i.p.)-injected mice, in standard aCSF (**G, H**) and gabazine (10 µM)-containing aCSF (**I, J**). Original recordings of electrically-evoked (e)EPSCs under both conditions (**G, I**). The stimulation artifacts were retouched from the recordings. eEPSCs were evoked by two stimuli (7 mA strength, 100 µs duration) with an inter-pulse interval of 50 ms, every 20 s. A438079 (10 µM) did not alter the P2/P1 ratio in the mean ± S.E.M. of 10 cells (**H**) in the absence of gabazine, although in a minority of cells there was a difference (**G;** n = 2) between preparations taken from saline- and KA-treated mice. There was no such variability (**I, J**) when gabazine blocked the GABA_A_R component of the eEPSC (n = 8). The statistical evaluation of data showed that the PPP in aCSF in comparison with that measured in aCSF + A438079, did not differ from each other in a statistically significant manner (**H, right panel**; t-test, t = 1.413). The same occurred in the presence of gabazine (**J, right panel**; t = 0.158).

Then, we turned to *ex vivo* electrophysiology to check modulations of agonist-induced transmembrane ionic currents in neurons and astrocytes of the hippocampal CA1 area by KA injection. For this purpose, hippocampal brain slices were prepared from KA- and solvent-treated mice, and NMDA (100 µM) at the beginning and end of each experiments, as well as Bz-ATP (1000-3000-1000 µM) spaced in between the NMDA responses, were applied every 3 min to the respective CA1 cells (Figure 9A-F). The evaluation of these experiments on astrocytes showed that KA injection preceding the preparation of the hippocampal slices by about 1 h did not alter the effect of Bz-ATP (1000 µM) in a statistically significant manner; there was no change on the next day either. However, the effect of Bz-ATP (3000 µM) currents in neurons appeared to increase 1 h after preparation, and this change became statistically significant on a second day. Hence, KA-treatment caused no potentiation of Bz-ATP currents after the induction of SE in astrocytes, but a statistically significant potentiation was observed in neurons one day following SE. At the same time the responses to NMDA in neurons were not changed by KA-injection (control: -843.6 ± 119.9 pA; 1 h: -584.9 ± 51.3 pA; 24 h: -1011.3 ± 181.4 pA; n = 8 each; one-way ANOVA, F_2-21_ = 2.522, P = 0.1044). These results indicate that, at least in neurons (in astrocytes there was no NMDA current at all), only the current responses to Bz-ATP were potentiated by KA-induced SE, while the NMDA induced current responses were not altered by the same treatment.

Our next task was to clarify whether endogenous ATP co-released with glutamate interacts postsynaptically in hippocampal CA1 neurons, or whether this ATP would presynaptically modulate glutamate release. This question had to be answered both under normal conditions and also in preparations taken from KA-treated mice. We stimulated with two pulses spaced apart by 50 ms, every 20 s for 20 min, in order to measure paired-pulse potentiation (PPP) as a P2/P1 ratio (Figure 9G-J). Hippocampal slices containing the CA1 area were again prepared 1 h after injecting solvent or KA to mice. Then, the CA1 neurons were whole-cell patch-clamped in a standard Ca^2+^/Mg^2+^-containing aCSF, and the P2/P1 ratio was determined. The increase of this ratio under superfusion with the P2X7R antagonist A438079 (10 µM) would indicate a presynaptic effect on transmitter release. However, this was not the case (Figure 9H). Under our experimental conditions, glutamate and GABA are released from separate nerve terminals and therefore, no change of electrically evoked postsynaptic current (ePSC) amplitudes signal opposite effects on glutamate and GABA secretion. To exclude the complicating influence of GABA, we included into the aCSF the GABA_A_R antagonist gabazine (10 µM). However, purely glutamate-mediated evoked excitatory postsynaptic currents (eEPSCs) still showed unchanged P2/P1 ratios, in spite of adding A438079 to the bath (Figure 9J).

A last complicating factor could, however, be eliminated by experiments carried out in low X^2+^ aCSF. It is well known that the P2X7R becomes much more sensitive to its agonists in the absence of Mg^2+^, and in a low Ca^2+^ concentration environment, compared to a normal Ca^2+^/Mg^2+^ aCSF. Therefore, we stimulated, in a low X^2+^ medium, the input to CA1 neurons with single pulses every 20 s for 20 min. The experimental protocol was as follows: we measured eEPSC amplitudes 5 min before, 10 min during, and 5 min after the application of A438079 (10 µM). Under these conditions there was no change of the eEPSC amplitudes by A-438079, in spite of using low X^2+^-aCSF. The solvent control recordings yielded the following eEPSC amplitudes (pre: -12.8±1.1 pA; A438079: -10.4±1.2 pA; post: -11.5±0.7 pA; n = 6 each; two-way ANOVA, Row-F_5,10_ = 09621, P = 0.4834, Column-F_2,10_ = 1.447, P = 0.2807) (n = 6). The corresponding eEPSC amplitudes, 1 h after the injection of KA (30 mg/kg, i.p.), were the following: pre: 11.5 ± 0.7; A438079: 13.1 ± 0.3; post: 12.4 ± 1.5; two way-ANOVA, Row-F_5,10_ = 0.4773, P = 07171, Column F_2,10_ = 05250, P = 0.6070) (n = 6). Hence, ATP co-released with glutamate/GABA modifies neither the postsynaptic effect of the transmitters, nor the presynaptic release mechanism.

### Electrophysiological measurement of pre- and postsynaptic effects of kainic acid-induced *status epilepticus* in hippocampal brain slices; spontaneous postsynaptic currents and spontaneous excitatory postsynaptic currents

In order to complete the ePSC and eEPSC experimental series, we also measured spontaneous (s)PSCs and sEPSCs in CA1 pyramidal neurons of the hippocampus. Figure S5A, B shows original recordings of sEPSCs (in the presence of 10 µM gabazine) in CA1 neurons of hippocampal slices, obtained from mice, which were treated with KA, 24 h before preparation of the brain slices.

We found that in hippocampal CA1 pyramidal neurons of KA-injected mice the sPSC frequency decreased, while the sPSC amplitude increased, on the first sight indicating a postsynaptic effect (Figure S5E, F). However, sPSCs are due to the combined and opposing effects of the excitatory transmitter glutamate and the inhibitory transmitter GABA, released simultaneously. Therefore, it somewhat astounded that in presence of the GABA_A_R antagonistic gabazine, the effect of KA-SE turned out to be purely pre-synaptic. Thus, KA did not influence in a statistically significant manner the sEPSC amplitude, while it massively facilitated the sEPSC frequency (Figure S5C, D). In consequence, the measurement of the nerve stimulation-evoked and spontaneous release of glutamate was affected in different manners by KA-induced *status epilepticus*. A possible explanation of this fact is that evoked transmitter release is completely dependent on extracellular Ca^2+^, in contrast to spontaneous release which is mostly dependent on intracellular Ca^2+^.

### Interaction of kainic acid-induced *status epilepticus* with long-term potentiation in hippocampal brain slices

**Figure 10:**
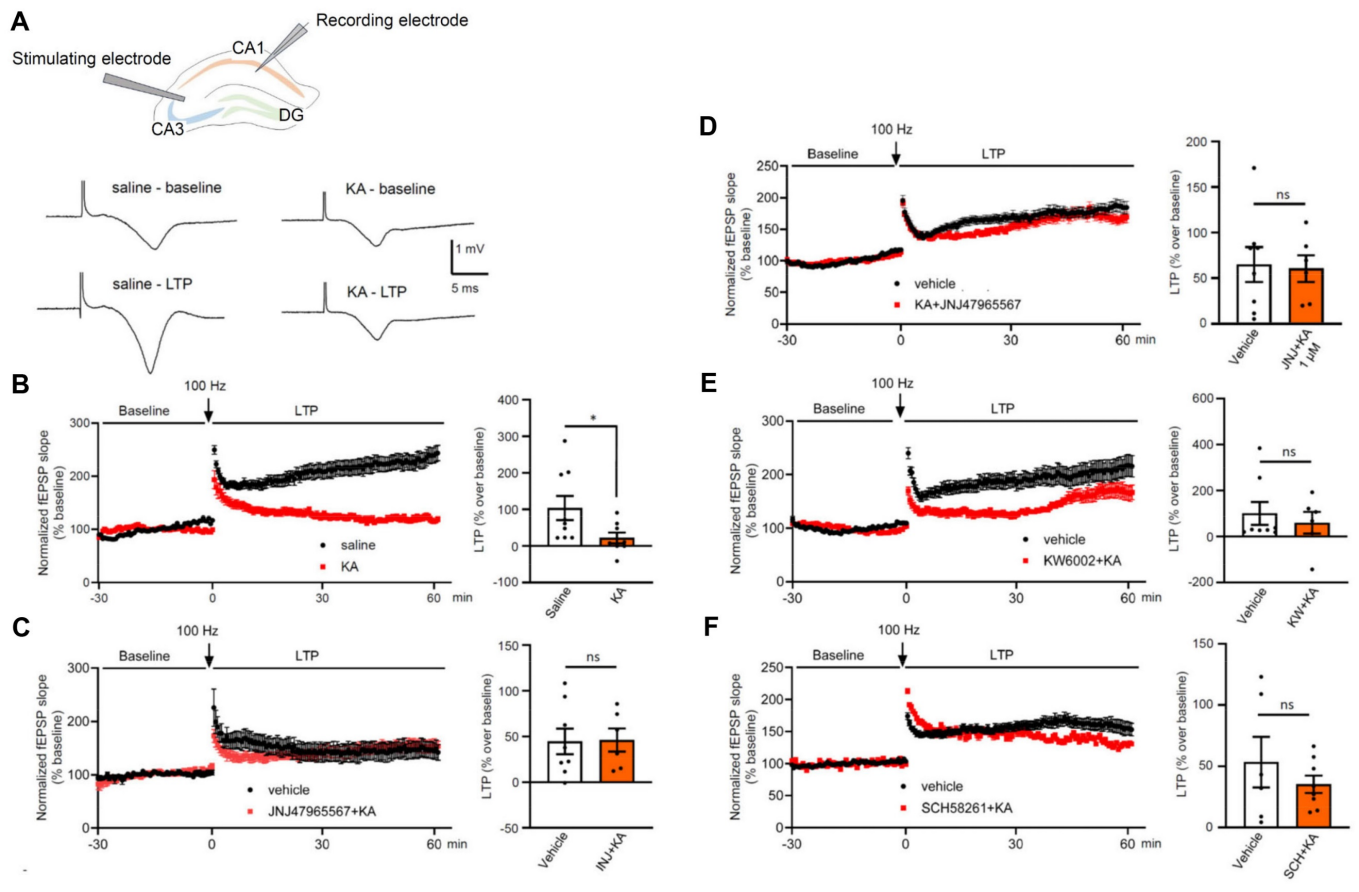
Kainic acid (30 mg/kg., i.p.) injection to mice blocks long-term potentiation (LTP) caused in hippocampal CA1 neurons by stimulation of the Schaffer collaterals. Field excitatory potentials (fEPSPs) were evoked in brain slice preparations by 100 µs duration square wave pulses every 20 s (stimulation strength, 7 mA). LTP was induced by high frequency stimulation (100 Hz for 1 s, repeated two times at an interval of 20 s). Experimental arrangement and original tracings in preparations taken from saline- and KA-treated mice (**A**). Electrophysiological measurement were made 24 h after injecting KA. The LTP was determined in the last 10 min of recording as a % over baseline value (**B-F**). (**B**) fEPSP slopes recorded for 30 min before and 60 min after high frequency stimulation in saline- and KA-injected animals. (**C**) Abolition of the effect of KA, when it was injected together with JNJ-47965567 (30 mg/kg, i.p.), on the fEPSP slope. (**D**) Abolition of the effect of KA injection when JNJ-47965567 (1 µM) was superfused onto brain slices. (**E, F**) Abolition of the effect of KA, when it was injected together with KW6002 or SCH58261 (3 mg/kg, i.p.) of mice before preparing brain slices. The unpaired t-test was used to calculate the levels of statistical significance in case of normal distribution; *P < 0.05, n.s. P > 0.05 (**B**, t = 2.205; **C**, t = 0.1701; **D**, t = 0.1099; **F**, t = 0.8476). The Man-Whitney test was used to calculate statistical significance in case of non-normal distribution; n.s. P > 0.05 (**E**, U = 23). The number of experiments varied between 6 and 9.

LTP (especially in the hippocampus) is generally considered to be a cellular model of learning and memory. Therefore, we applied high frequency stimulation to the Schaffer collaterals projecting to the CA1 neurons and recorded a long-lasting increase of the slopes of electrically-evoked field excitatory postsynaptic potentials (fEPSP) (Figure 10A). The experimental arrangement was the following: control fEPSPs were recorded for 30 min, then two high frequency stimulation periods were delivered at 100 Hz each, and afterwards the fEPSPs were recorded for another 1 h (see Methods). This schedule was adopted to hippocampal slices prepared from mice injected the day before with saline or KA, the latter preceded by the injection of vehicle or a P2X7/A2AR antagonist (Figure 10B-F). The control application of saline resulted in the expected long-lasting increase of the fEPSP slope (LTP), which significantly surmounted the negligible change observed after KA treatment (Figure 10B). The LTP was determined in the last 10 min of recording as a percentage over baseline (Figure 10B-F). The injection of the P2X7R antagonist JNJ-47965567 (30 mg/kg, i.p.) before KA prevented the inhibition of the LTP caused by KA (Figure 10C). Interestingly, superfusion of hippocampal slices with JNJ-47965567 (1 µM), prepared from KA injected mice had the same effect on the LTP (Figure 10D). Accordingly, two A2AR antagonists, KW6002 and SCH58261 (3 mg/kg each, i.p.), when they were injected before KA the day before the preparation of brain slices, also prevented the inhibitory effect of KA on the normal LTP amplitude (Figure 10E, F). We conclude, that this *ex vivo* study modelled and corroborated our *in vivo* results, demonstrating that KA-SE interfered with LTP induction (and also cognitive abilities), and stimulation of either P2X7Rs or A2ARs by released ATP and adenosine, respectively, mediated this effect.

## Discussion

From the three standard chemogenic stimuli evoking SE (KA, pilocarpine, pentylenetetrazole), we have chosen the first one because the KA induced seizure lesions (and therefore the assumed primary sites of action) are mainly located on the edge of the hippocampus, perfectly mimicking the classical histopathological features of hippocampal sclerosis, the hallmark of temporal lobe epilepsy 41. There is also a general consensus that the hippocampus is a structure frequently involved in human acquired epilepsy, and mesial temporal lobe epilepsy in particular is characterized by resistance to therapy 42,43. One of the main findings of our experiments is the sharp and immediate rise of Ca^2+^ signals in the hippocampal CA1 region. It is most interesting, that this rise occurred mostly via P2X7R activation both in neurons and astrocytes to a comparable extent; the preceding injection of the highly selective and brain permeable P2X7R antagonist JNJ-47965567 considerably inhibited the increase in Ca^2+^ signaling; the A2AR antagonists KW6002 and SCH58261 had the same effect. The CA1 area seems to be important for ictogenesis and probably also epileptogenesis, and P2X7/A2ARs seem to be significant regulators of these pathological phenomena, so the ATP-P2X7R / adenosine-A2AR axis may be a contributor/regulator of seizures.

Recently, there has been extensive discussions whether P2X7Rs are located both at neuronal and non-neuronal cells of the CNS, or only at the non-neuronal ones (astrocytes, oligodendrocytes; 39,40), releasing signaling molecules (e.g. glutamate) onto neighboring neurons 32,44. A similar indirect effect may also take place in our present experiments, although, because of methodological reasons, we did not investigate the influence of antagonists of e.g. ionotropic glutamate receptors on the KA-SE-induced increase in neuronal/astrocytic Ca^2+^ signaling. Nevertheless, we did investigate the effect of cell-specific P2X7R-shRNA pre-treatment on the KA-SE-induced deterioration of cognitive performance of mice in the MWM test system, and found that the abrogation of P2X7Rs in hippocampal CA1 astrocytes, but not neurons prevented this deterioration. Therefore, our experiments support the involvement of astrocytic rather than neuronal P2X7Rs in the observed effects (a detailed discussion on the cognitive impairment by SE, see later).

The KA- or pilocarpine-induced seizures in rats 45,46 and mice 47,48 led to an increased P2X7R immunolabelling in neocortical neurons; the same increase was detected in the brain samples from epileptic patients in comparison with the post-mortem brains of humans with no previous history of neurological disease 49. Although the correctness of these findings is not questioned at all, the insecurity inherent with the use of polyclonal antibodies raised against P2X7Rs has been pointed out repeatedly 50. More recent data cast doubt on the neuronal localization of P2X7Rs, and suggest an exclusively glial localization 51, albeit some results still continue to support the neuronal one 23,52.

Similarly, the increased density of the A2AR-protein was observed both in amygdala-kindled rats and following systemic KA-induced SE 16,53 or early-life hyperthermic seizures 54. In addition, there was a threefold increase in A2ARs and also the adenosine producing enzyme ecto-5'-nucleotidase (CD73) in hippocampal astrocytes of patients with mesial temporal lobe epilepsy, when compared to controls 55. The ecto-5'-nucleotidase, generating adenosine from ATP, and thereby indirectly targeting A2ARs, was also up-regulated after KA-SE in rats 11, in complete accordance with our results in mice.

The vesicular and Ca^2+^-dependent release of ATP has been recognized both in neurons 56,57, and to a minor extent also astrocytes 58,59. In addition, ATP may leave the cytoplasm of astrocytes also by non-vesicular mechanisms, through ATP permeable channels (connexin hemichannels, pannexin-1 channels, calcium homeostasis modulator 1, volume-regulated anion channels, maxi-anion channels and the bestrophin-1 channel 59,60. Connexin hemichannels may release besides ATP also glutamate/GABA 61, and Na^+^-dependent high-affinity glutamate versus GABA uptake could result in a differential enrichment of the two transmitters within the perisynaptic region of neurons 62,63).

ATP is enzymatically degraded by ectonucleoside triphosphate diphosphohydrolase (CD39) to AMP and then by ecto-5'-nucleotidase (CD73) to adenosine 7,64. Besides this 'conventional' and Ca^2+^-dependent generation of adenosine, the nucleoside may be also outpoured from the intracellular space of astrocytes by the operation of the equilibrative nucleoside transporter 2 (ENT-2; 65). In our experiments, the slow time-course and low quantity of adenosine released together with ATP suggests either an exocytotic release of adenosine 66 or a Ca^2+^-dependent operation of an equilibrative nucleoside transporter 67 as a mechanisms for the accumulation of adenosine following ATP release.

Although multiple reports confirm that ATP/adenosine are released under *in vitro* conditions from brain slices/cell culture systems/synaptosomes by modelling epileptic seizures, there is a paucity of convincing evidence for a similarly increased release of the two purines in the CNS, during *in vivo* seizure activity 10,67. Hence, our own experiments with the use of genetic probes are of considerable significance to support the long-standing assumption that SE triggers ATP/adenosine release in the hippocampus.

After having recognized the P2X7 and A2AR-sensitive hippocampal increase of Ca^2+^ signaling as a consequence of KA-induced SE, we asked ourselves, whether cognitive limitation can be observed after SE, and whether this response is due to the stimulation of the above receptors. The Morris Water Maze is a test for investigating hippocampal-dependent spatial memory 68 and the Novel Object Recognition measurement a test for studying hippocampal-dependent non-spatial memory 69. We used these tests and found that the activation of P2X7 and A2ARs equally compromised the learning abilities of mice after permanent seizures caused by KA injection.

Cognitive deterioration is a well-known implication of P2X7R stimulation, which is due to two possibly independent effects. (1) The enhanced production of cytokines (in the first line that of IL-1β) is an obligatory response following the activation of P2X7Rs. While IL-1β, IL-6 and TNFα were found to support long-term plasticity and learning/memory processes under physiological conditions; during inflammation, the effects of elevated cytokine levels are mostly detrimental to memory mechanisms 70,71. Hence, P2X7R blockade might decrease the duration of SE and improve the consequent cognitive abilities, which was the case in our experiments with the pre-application of a respective antagonist. (2) A further explanation for the negative effect of P2X7R activation on hippocampal mnemonic processes is necrosis/apoptosis of radial glia-like adult NPCs in the hippocampal subgranular zone 72. Mature granule cells of the hippocampal dentate gyrus are generated both during development and adult neurogenesis, owing to their high activation threshold and input specificity, they serve as pattern separators 73. The P2X7R-mediated damage of NPCs is certainly not involved in the cognitive impairment developing within 24 h, but may have a role in such a process on a longer-lasting time-scale.

The subgranular zone of the hippocampal dentate gyrus is one of the neurogenic niches harboring adult doublecortin-immunoreactive NPCs 72,74. In the present experiments we made use of the doublecortin-immunopositivity of NPCs and the fact that temporal lobe epilepsy (and its rodent model, KA-SE) is known to cause a prolonged increase in dentate NPC proliferation 75,76. An obligatory long-term consequence of temporal lobe epilepsy is a co-morbidity with cognitive dysfunction 77, which is due to an increased number of functionally disturbed NPCs, displaying hilar basal dendrites with spines, ectopic hilar localization of the cells bodies, and mossy fiber sprouting 78,79. Since, we found that the genetic deletion of P2X7Rs led after 3 weeks to a moderate facilitation of the KA-SE-induced number of NPCs when compared with their WT counterparts, it was concluded that in WT mice there is a permanent, although minor control of NPC numbers by P2X7R activity. These results perfectly correlate with the assumption that functionally intact NPCs are needed for undisturbed cognitive achievements.

Adenosine A2AR activation modulates learning and memory processes in mice, for instance striatopallidal A2ARs exert an inhibitory control on a variety of cognitive functions, ranging from working memory 80, to goal-directed behavior 81, motor sequence learning 82, strategy shifting 83, and decision making 84. These results in association with the finding that adenosine release is boosted by SE, strongly suggest that A2ARs are involved in the SE-induced worsening of memory performance in mice. Inhibition of adenosine receptors in zebrafish by A1 and A2AR selective antagonists equally prevented memory disturbance after pentylenetetrazole-induced seizures 85.

As already mentioned, hippocampal sclerosis is either a cause or a consequence of epilepsy, contributing to generation of epileptic seizures 42,43. Considering that major depression was identified as a prominent co-morbidity of epilepsy, it is worth noting that the medial prefrontal cortex, involved in emotional or affective functions, is considered to be one of the depression-relevant areas of the brain 86. Therefore, we measured the effect of KA on Ca^2+^ signaling in mPFC neurons and astrocytes, and found in both cases an increase. Although the P2X7R antagonist JNJ-47965567, just as in the hippocampal CA1 region, reduced the observed increase of Ca^2+^ signals, KA-SE had no consequences on depressive-like reactions measured in the TST, FST and SPT. Prolongation of immobility in TST and FST of mice are considered as indicators of behavioral despair 87, and a decrease in SCT, as an indicator of anhedonic behavior 88, typical for a depressive-like state. Similarly, the time spent in the center of the open-field apparatus, which is considered to be a measure of the anxiolytic-depressive state of mice, was not altered by JNJ-47965567, either.

To identify the CA1 pyramidal cells as primary regulators of epileptic activity after the injection of KA, we prepared hippocampal brain slices and recorded Bz-ATP and NMDA-induced membrane currents from these cells and also from the neighboring astrocytes. In contrast to the astrocytes, pyramidal neurons exhibited much larger current responses to Bz-ATP in preparations taken after both 1 h and 24 h after KA-injection. Although originally, pathological neuronal functions were supposed to be the exclusive drivers of epileptic seizures, more recently glial cells were suggested to play significant roles in SE through glia-neuron interactions 89-91. The present experiment showed a prominent and selective excitability increase in response to the P2X7R prototypic agonist Bz-ATP in CA1 pyramidal cells - possibly by stimulating nearby microglia/oligodendrocytes, which release a hitherto unidentified transmitter/signaling molecule onto neurons 32,39.

After finding out that KA-treatment of mice causes a P2X7R-mediated excitability increase of CA1 pyramidal cells, we tested whether this effect was pre- or postsynaptic. A presynaptic effect would result in the facilitation of glutamate release, while the postsynaptic effect would result in a sensitivity increase mediated by P2X7Rs located at the pyramidal cell membrane. In our experiments, paired-pulse potentiation of glutamate-mediated eEPSCs remained unchanged in the presence of the P2X7R antagonist A-438079, indicating that a postsynaptic site of action prevails. Moreover, eEPSC amplitudes caused by single pulse stimulation, but in a low X^2+^ medium, were also not modified by A-438079.

Long-term potentiation is a cellular model of learning and memory 92. Brief high frequency stimulation of hippocampal excitatory synapses produced a rapid and long-lasting increase in the strength of these synapses that could persist for many days. Many types of LTP exist, but the classic one is that which occurs in the hippocampus, depends on NMDAR activation, and primarily involves a postsynaptic modification. P2X7Rs were reported to be involved in gliogenic LTP in the rat spinal cord, induced by glial cell activation and mediated by diffusible second-messengers, such as D-serine and TNFα 93,94. Furthermore, A2ARs were also found to participate in LTP-induction in the rat hippocampus and exhibited age-dependent changes, with a larger predisposition in young animals 95.

In conclusion, KA-induced SE increased Ca^2+^ signaling in CA1 pyramidal neurons and astrocytes as determined by fiber photometry (Figure S6). Increased Ca^2+^ signaling is a measure of the rise in [Ca^2+^]_i_. This probably led to the release of glutamate from neurons and astrocytes stimulating glutamate ionotropic receptors of the NMDA and AMPA types at neighboring CA1 neurons. The stimulation of these receptors induces LTP, considered as a cellular basis of learning/memory. Simultaneously, ATP is also released from both CA1 neurons and astrocytes. This ATP activates P2X7 receptors at CA1 astrocytes and possibly also neurons, inhibiting indirectly or directly the hippocampus-dependent cognitive processes. Thus, SE appeared to induce an increase of [Ca^2+^]_i_ in the major cell types of the hippocampus. This caused the release of ATP, the stimulation of P2X7Rs, and in consequence cognitive deficits. We also found that adenosine co-released with ATP or generated by its enzymatic degradation contributed to the deterioration of cognitive abilities by stimulating hippocampal A2ARs.

We applied the P2X7 and A2AR antagonists always before the ATP and adenosine-releasing KA-SE, according to the usual protocol in pharmacological analyses. The sequence of the ligand applications was the following: at first an antagonist was administered, and then, in its continuing presence, the corresponding agonist. In fact, we did not investigate whether a manifest KA-SE can be relieved by the subsequent application of P2X7 or A2AR antagonists. We considered this clinically important question beyond the scope of the present work, and a subject for future studies.

## Supplementary Material

Supplementary figures.

## Abbreviations

[Ca^2+^]_i_: intracellular Ca^2+^ concentration
AUC: area under the curve
Bz-ATP: Dibenzoyl-ATP
CA1: *cornu ammonis 1*
CD39: ectonucleoside triphosphate diphosphohydrolase
CD73: ecto-5'-nucleotidase
EGFP: enhanced green fluorescent protein
EPSC: excitatory postsynaptic current
FST: Forced Swim test
GFAP: glial fibrillary acidic protein
IL-1β: interleukin-1β
KA: kanic acid
mPFC: medial prefrontal cortex
MWM: Morris Water Maze
NPC: neural progenitor cell
NLRP3: inflammasome
NORT: Novel Object Recognition test
R: receptor
SE: *status epilepticus*
SPT: Sucrose Preference test
TLR4: toll-like receptor 4
TNFα: tumor necrosis factor α
TST: Tail Suspension test
