# Supplementary material for: Hippocampal P2X7 and A2A purinoceptors mediate cognitive impairment caused by long-lasting epileptic seizures
Source: Theranostics. 2025 Feb 10;15(7):3159–84. doi: 10.7150/thno.100365 (PMC11898279; doi:10.7150/thno.100365)
Supplement: Supplementary file 1 — Supplementary figures. [file thnov15p3159s1.pdf]

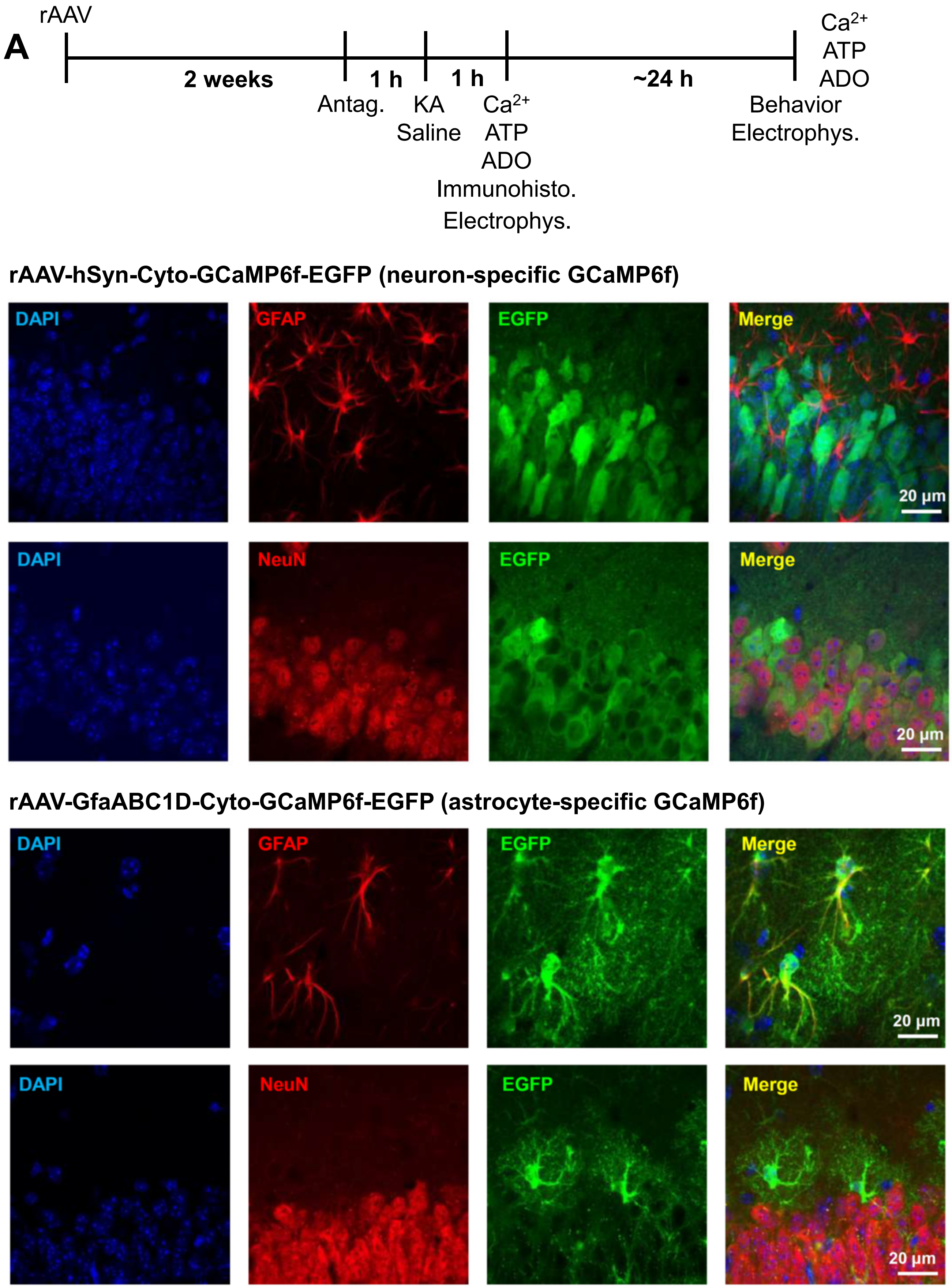
A
rAAV
2 weeks
1 h
1 h
~24 h
Antag.
KA
Saline
Ca^{2+}
ATP
ADO
Immunohisto.
Electrophys.
Behavior
Electrophys.
Ca^{2+}
ATP
ADO
rAAV-hSyn-Cyto-GCaMP6f-EGFP (neuron-specific GCaMP6f)
B
DAPI
GFAP
EGFP
Merge
20 µm
C
DAPI
NeuN
EGFP
Merge
20 µm
rAAV-GfaABC1D-Cyto-GCaMP6f-EGFP (astrocyte-specific GCaMP6f)
D
DAPI
GFAP
EGFP
Merge
20 µm
E
DAPI
NeuN
EGFP
Merge
20 µm

Figure S1 Time-dependent schedule of the experiments and immunohistochemical verification of the correct synthesis of the genetic Ca^{2+} indicator GCaMP6f in the targeted cells of the hippocampus. (**A**) Experimental schedule with the time-intervals shown. (**B**) No co-staining of the neuronal marker NeuN with EGFP. (**C**) Co-staining of the neuronal maker NeuN and EGFP (proving the presence of neuron-specific GCaMP6f) in CA1 hippocampal neurons of the mouse. (**D**) Co-staining of the astrocytic marker GFAP with EGFP (proving the presence of the astrocyte-specific GCaMP6f) in CA1 hippocampal astrocytes of the mouse. (**E**) No co-staining of the astrocytic marker GFAP with EGFP. It was not possibly to quantitatively evaluate these experiments, because neurons and astrocytes in the CA1 region were very densely populated and could not be easily discerned from each other in our specimens.

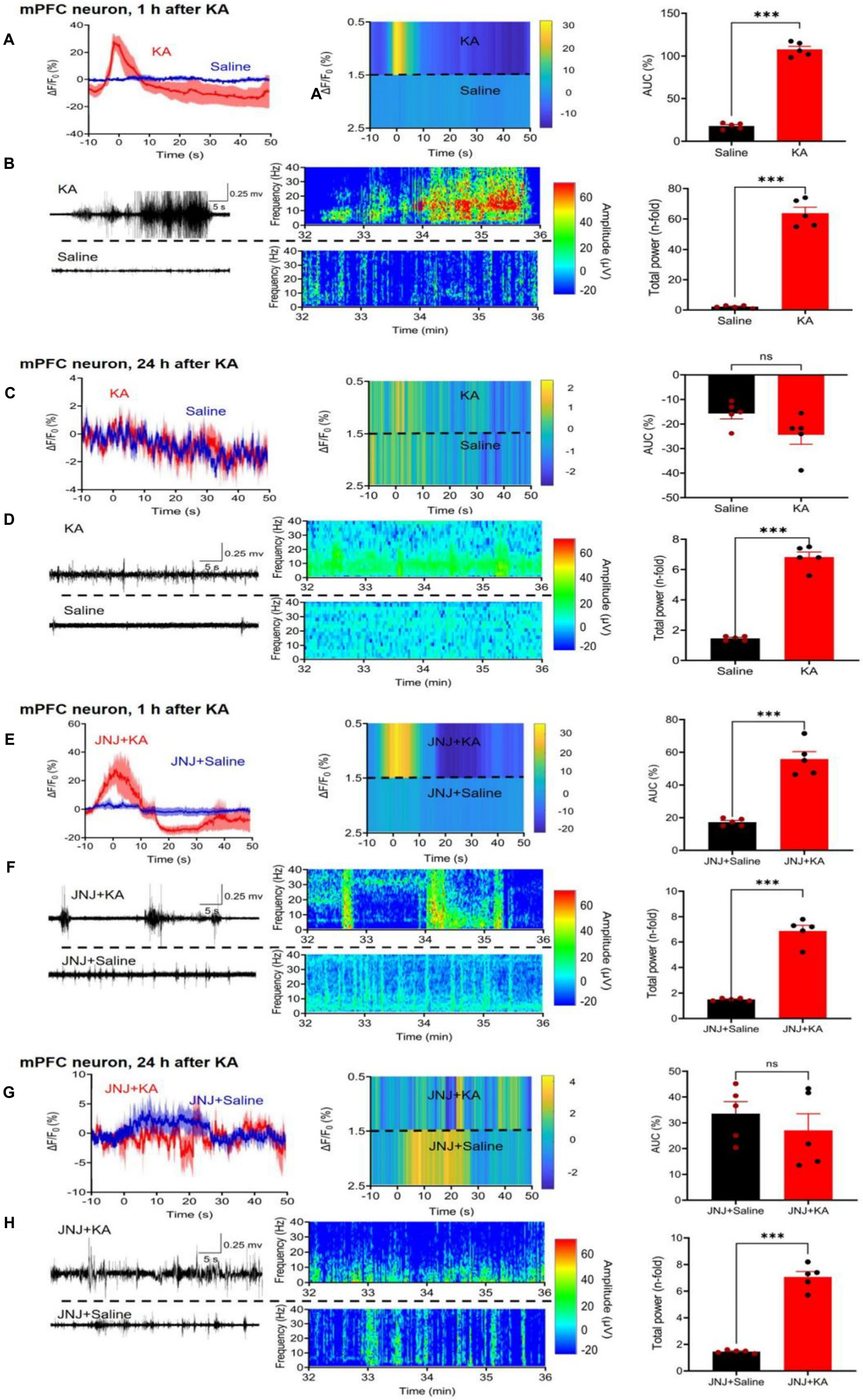

mPFC neuron, 1 h after KA
A
KA
Saline
ΔF/F0 (%)
Time (s)
AUC (%)
***
B
0.25 mv
5 s
Frequency (Hz)
Amplitude (μV)
Time (min)
Total power (n-fold)
mPFC neuron, 24 h after KA
C
ns
D
mPFC neuron, 1 h after KA
E
JNJ+KA
JNJ+Saline
F
mPFC neuron, 24 h after KA
G
H

Figure S2 Effect of kainic acid (KA) injection alone or in combination with the P2X7R antagonist JNJ-4438079 on Ca^{2+} signals in mPFC neurons of the mouse hippocampus. Two weeks before KA application (30 mg/kg, i.p.), the virus complex rAAV-hSyn-GCaMP6f-EGFP generating specifically in neurons a genetic Ca^{2+} indicator was microinjected into the hippocampus. JNJ-47965567 (30 mg/kg, i.p.) was injected 1 h before KA. The changes in Ca^{2+} signaling were measured with fiber photometry and expressed in $\Delta F/F_0$, and gross electrical activity of the brain was measured with EEG telemetry and presented in μV amplitude signals (left panels). The $\Delta F/F_0$ fluorescence curves are shown as mean ± S.E.M. of the recordings occurring during the seizure stages 4 and 5. KA was dissolved in saline, while JNJ-47965567 was dissolved in 30% SBE-β-CD+70% saline (the solvent of JNJ-4438079 is indicated in the Figure panels as saline for the sake of simplicity). All details of the procedures used are described in the Methods Section. EEG recordings are shown in the middle panels, while the right panels show the area under the curve (AUC) of the $\Delta F/F_0$ changes and the total power of the EEG as n-time changes from the baseline. The levels of statistical significance reached are marked with *P < 0.05, **P < 0.01, ***P < 0.001, n.s. P > 0.05. The unpaired t-test was used for evaluation throughout (**A**, t = 22.27; **B**, t = 15.51; **C**, t = 1.940; **D**, t = 15.54; **E**, t = 8.256; **F**, t = 12.05; **G**, t = 0.8166; **H**, t = 13.38). The number of experiments was 5 in each panel.

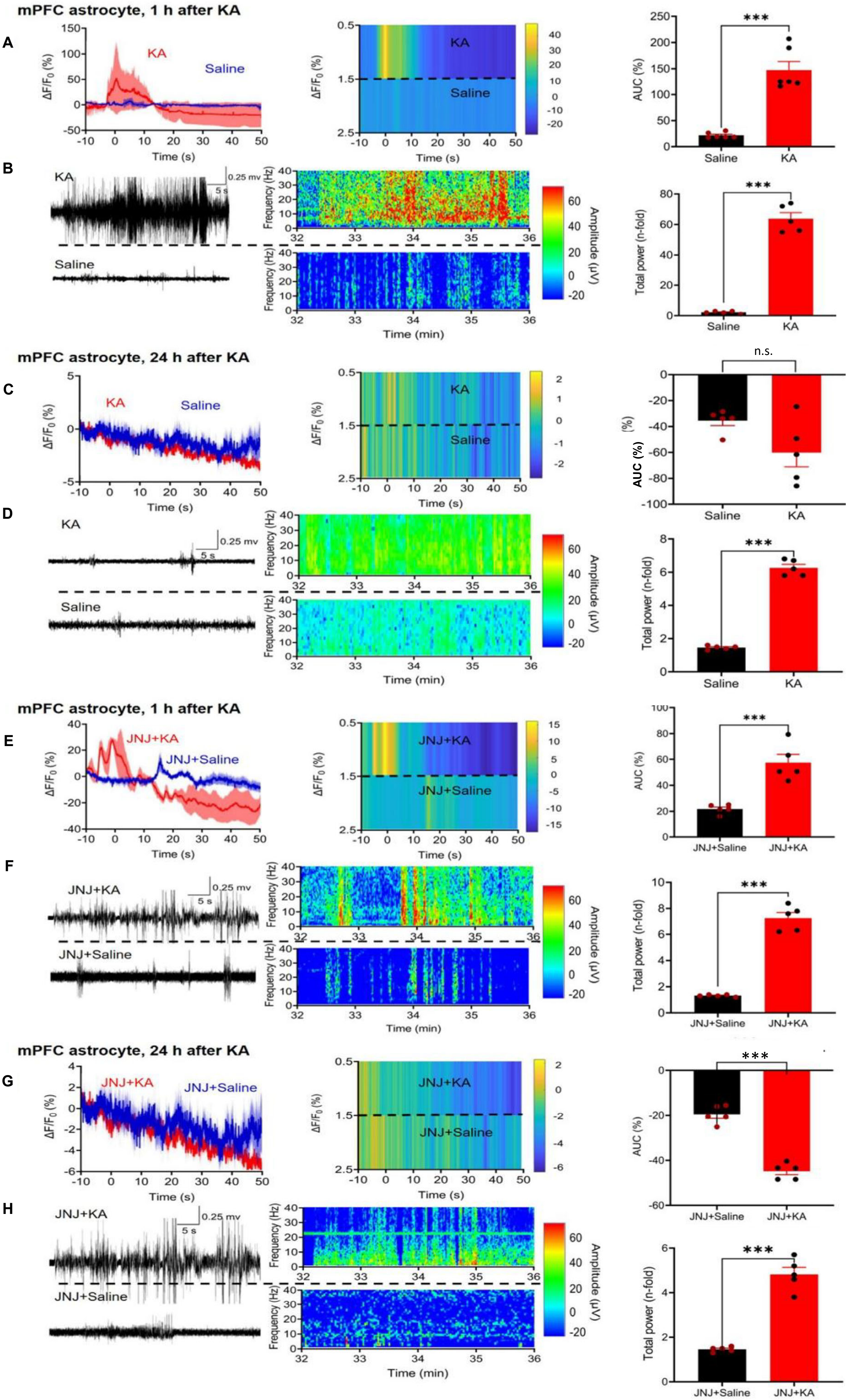

mPFC astrocyte, 1 h after KA
A
KA
Saline
ΔF/F0 (%)
Time (s)
AUC (%)
***
B
KA
Saline
0.25 mv
5 s
Frequency (Hz)
Time (min)
Amplitude (μV)
Total power (n-fold)
***
mPFC astrocyte, 24 h after KA
C
KA
Saline
ΔF/F0 (%)
Time (s)
AUC (%)
n.s.
D
KA
Saline
0.25 mv
5 s
Frequency (Hz)
Time (min)
Amplitude (μV)
Total power (n-fold)
***
mPFC astrocyte, 1 h after KA
E
JNJ+KA
JNJ+Saline
ΔF/F0 (%)
Time (s)
AUC (%)
***
F
JNJ+KA
JNJ+Saline
0.25 mv
5 s
Frequency (Hz)
Time (min)
Amplitude (μV)
Total power (n-fold)
***
mPFC astrocyte, 24 h after KA
G
JNJ+KA
JNJ+Saline
ΔF/F0 (%)
Time (s)
AUC (%)
***
H
JNJ+KA
JNJ+Saline
0.25 mv
5 s
Frequency (Hz)
Time (min)
Amplitude (μV)
Total power (n-fold)
***

Figure S3 Effect of kainic acid (KA) injection alone or in combination with the P2X7R antagonist JNJ-4438079 on Ca^{2+} signals in mPFC astrocytes of the mouse hippocampus. Two weeks before KA application (30 mg/kg, i.p.), the virus complex rAAV-GfaABC1D-GCaMP6f-EGFP generating specifically in astrocytes a genetic Ca^{2+} indicator was microinjected into the hippocampus. JNJ-47965567 (30 mg/kg, i.p.) was injected 1 h before KA. The changes of Ca^{2+} signals were measured with fiber photometry and expressed in $\Delta F/F_0$ and gross electrical activity of the brain was measured with EEG telemetry and presented in μV amplitude signals (left panels). The $\Delta F/F_0$ fluorescence curves are exhibited as mean ± S.E.M. of the recordings occurring during the seizure stages 4 and 5. KA was dissolved in saline, while JNJ-47965567 was dissolved in 30% SBE-β-CD+70% saline (the solvent of JNJ-4438079 is indicated in the Figure panels as saline for the sake of simplicity). All details of the procedures used are described in the Methods Section. EEG recordings are shown in the middle panels, while the right panels show the area under the curve (AUC) of the $\Delta F/F_0$ changes and the total power of the EEG as n-time changes from the baseline. The levels of statistical significance reached are marked with $^*P < 0.05$, $^{**}P < 0.01$, and $^{***}P < 0.001$, n.s. $P > 0.05$. The unpaired t-test was used for evaluation throughout (**A**, t = 6.693; **B**, t = 13.81; **C**, t = 0.2122; **D**, t = 21.86; **E**, t = 5.460; **F**, t = 13.66; **G**, t = 10.72; **H**, t = 10.47). The number of experiments was 5-6 in each panel.

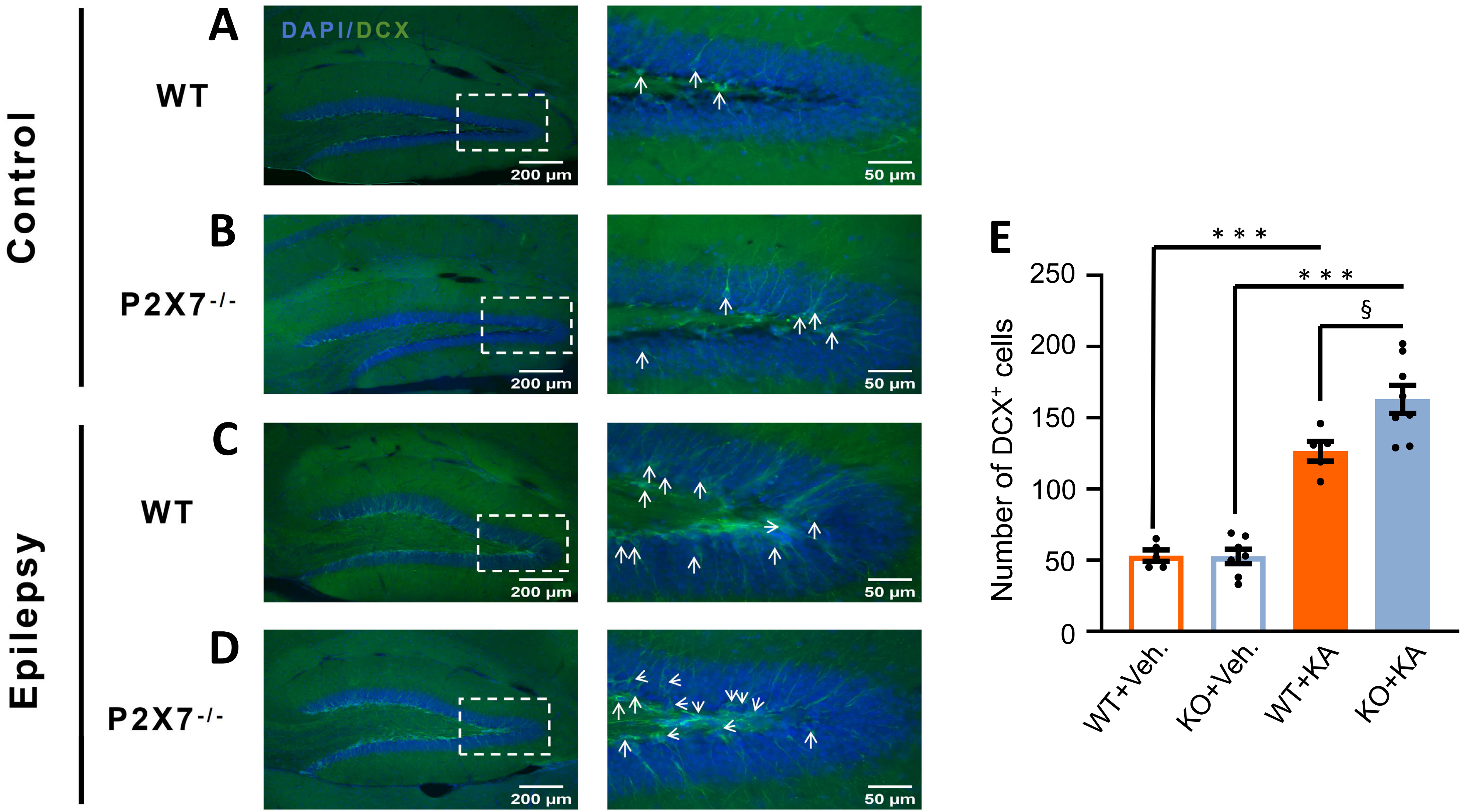
Control
Epilepsy
A
B
C
D
WT
P2X7-/-
WT
P2X7-/-
DAPI/DCX
200 µm
50 µm
E
Number of DCX+ cells
250
200
150
100
50
0
***
***
§
WT+Veh.
KO+Veh.
WT+KA
KO+KA

Figure S4 Measurement of the number of doublecortin (DCX)-immunopositve adult neural progenitor cells (NPCs) in the subgranular zone of the dentate gyrus both in the hippocampus of wild-type (WT) and P2X7R knockout ($P2X7^{-/-}$) mice. Representative immunohistochemistry specimens of WT and epileptic mice (**A-D**); *status epilepticus* (epilepsy) was initiated by the intraperitoneal injection of 30 mg/kg kainic acid (KA). Preparation of the slices and their staining was made 3 weeks after saline or KA injection. Laser scanning microscopy helped to achieve the needed large magnifications. The right set of panels in a row (**A-D**) shows the area of the hippocampus boxed in the left set of panels at a higher magnification. The white arrows point to individual NPCs. The genetic deletion of the P2X7R resulted in a higher number of DCX-immunopositive cells in P2X7R KO than in wild-type (WT) mice. Mean ± SEM of 5-8 cells from 3 mice are included in each column (**E**). One way ANOVA was used for statistical evaluation. The levels of statistical significance reached are marked with ***$P < 0.001$, or $^{\S}P < 0.05$ ($F_{3,21} = 55.00$, $P < 0.0001$; Tukey's test, WT+Veh. vs. WT+KA, $P < 0.0001$; KO+Veh. vs. KO+KA, $P < 0.0001$, WT+KA vs. KO+KA, $P < 0.0165$). Veh., vehicle (saline).

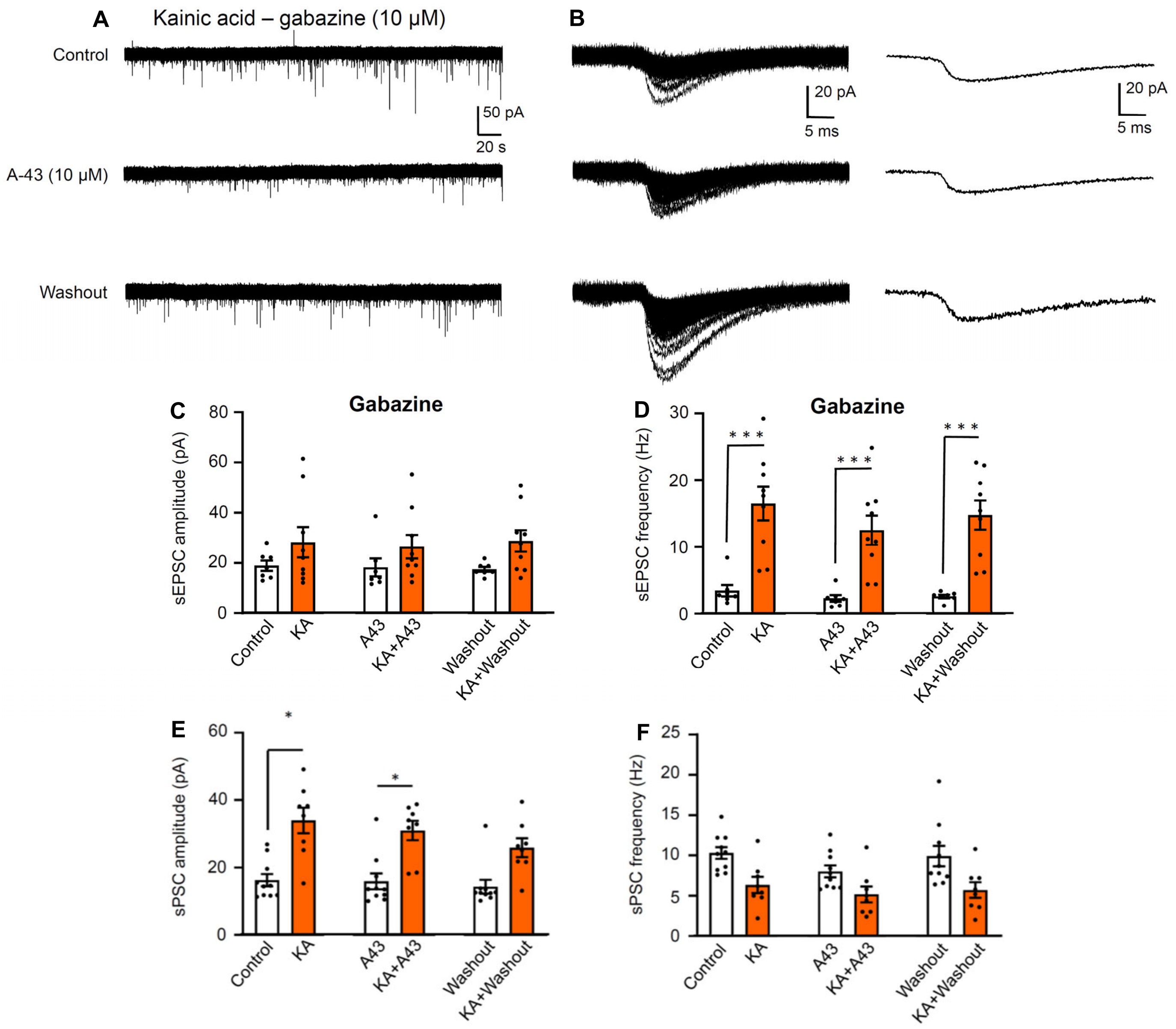
A
Kainic acid – gabazine (10 µM)
Control
A-43 (10 µM)
Washout
50 pA
20 s
B
20 pA
5 ms
20 pA
5 ms
C
Gabazine
sEPSC amplitude (pA)
0
20
40
60
80
Control
KA
A43
KA+A43
Washout
KA+Washout
D
Gabazine
sEPSC frequency (Hz)
0
10
20
30
* * *
* * *
* * *
Control
KA
A43
KA+A43
Washout
KA+Washout
E
sPSC amplitude (pA)
0
20
40
60
*
*
Control
KA
A43
KA+A43
Washout
KA+Washout
F
sPSC frequency (Hz)
0
5
10
15
20
25
Control
KA
A43
KA+A43
Washout
KA+Washout

Figure S5 Spontaneous postsynaptic currents (sPSC) and spontaneous excitatory postsynaptic currents (sEPSCs) in CA1 pyramidal neurons in hippocampal brain slices taken from mice injected with solvent or kainic acid (KA 30 mg/kg, i.p.) 1 h before preparation. sPSCs are due to responses to the spontaneous release of glutamate and GABA, while sEPSCs are due to responses of glutamate only in the presence of the $GABA_AR$ antagonist gabazine (10 µM). sPSCs and sEPSCs were recorded for 5 min, before superfusion with the P2X7R antagonist A438079 (A43), during a 5-min superfusion with this antagonist, and 5 min after its washout. Representative recordings of sEPSCs (**A**) and superimposed sEPSCs as well as their means (**B**) in KA-treated preparations. sEPSC amplitudes (**C**) and their frequency (**D**) in a mean±SEM of 7-9 cells from 4-5 mice. sPSC amplitudes (**C**) and their frequency (**D**) in a mean±SEM of 8-9 cells from 4-5 mice. One way ANOVA (**C, D**) or Kruskal-Wallis ANOVA on ranks (**E**, **F**) was used for statistical evaluation. The levels of statistical significance reached are marked with ***P < 0.0001, *P < 0.05 (**D**, $F_{7,58} = 20.34$, P < 0.0001; Tukey's test, Control vs. KA, P < 0.0001, A43 vs. KA, P < 0.0008, Washout vs. KA+Washout, P < 0.0001; **E**, Kruskal-Wallis statistics = 28.24, P < 0.0001; Tukey's test. Control vs. KA, P = 0.0215, A43 vs. KA, P = 0.0315).

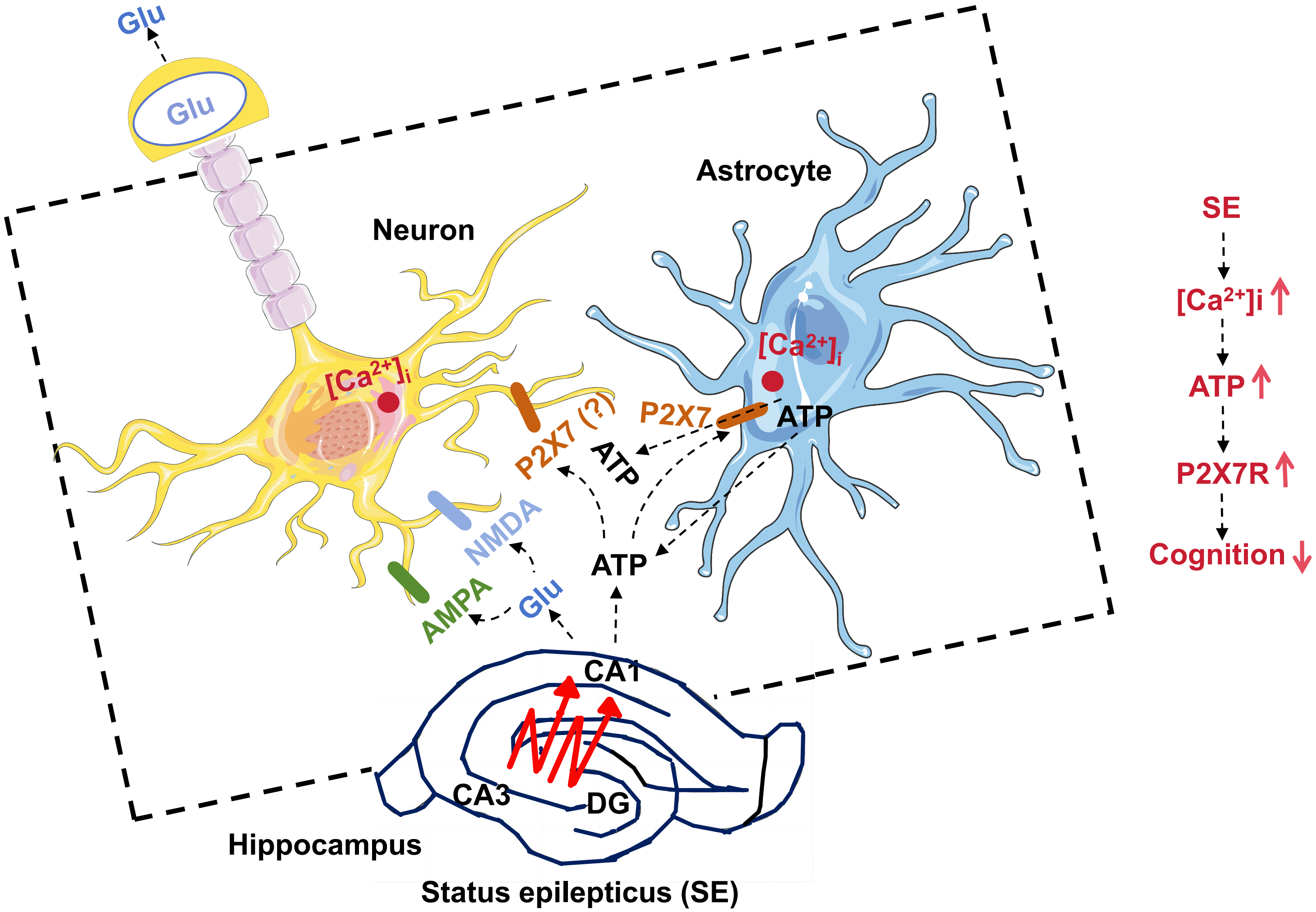

Glu
Glu
Neuron
Astrocyte
[Ca2+]i
[Ca2+]i
P2X7 (?)
P2X7
ATP
ATP
ATP
NMDA
AMPA
Glu
CA1
CA3
DG
Hippocampus
Status epilepticus (SE)
SE
[Ca2+]i ↑
ATP ↑
P2X7R ↑
Cognition ↓

Figure S6 Kainic acid-induced *status epilepticus* (SE) increased the intracellular Ca^{2+} concentration in CA1 pyramidal neurons and astrocytes. This triggered a sequence of events leading to cognitive limitation via the activation of P2X7Rs, as described in the Discussion Section of this article. For easier understanding, only the involvement of ATP in this process in exemplified; however, the outflow of adenosine (generated by the enzymatic degradation of ATP, or probably released on its own right) also causes similar effects on the cognitive abilities by stimulating adenosine A2ARs. CA1, cornu ammonis 1; CA3, cornu ammonis 3, DG, dentate gyrus.
